# Face reverse degree topological analysis of TP-COFs, existence of isentropic COFs and spectral characteristics

**DOI:** 10.3389/fchem.2026.1823507

**Published:** 2026-06-17

**Authors:** Thirsha Rajendran, Micheal Arockiaraj, Arul Jeya Shalini, Huda M. Alshanbari, Nawal Al-Hoshani

**Affiliations:** 1 Department of Mathematics, Loyola College, University of Madras, Chennai, India; 2 Department of Mathematics, Loyola College, Chennai, India; 3 Department of Mathematics, Women’s Christian College, Chennai, India; 4 Department of Mathematical Sciences, College of Science, Princess Nourah bint Abdulrahman University, Riyadh, Saudi Arabia; 5 Department of Biology, College of Science, Princess Nourah bint Abdulrahman University, Riyadh, Saudi Arabia

**Keywords:** face reverse degree indices, HOMO‐LUMO gap, isentropic structures, QSPR modeling, spectral energy, triple-pore covalent organic frameworks

## Abstract

The recent synthesis of triple-pore covalent organic frameworks (TP-COFs), specifically TP-COF-DAB and TP-COF-BZ, represents a significant milestone in framework chemistry, as replicating such multi-pore architectures remains highly challenging. Despite their structural novelty, the mathematical perspective of their topology and associated properties is still limited. In this study, closed-form expressions are derived for reverse degree topological indices of these TP-COFs based on a bitrapezium topological arrangements. Scaled face reverse degree indices are employed to explicitly characterize pore geometry in triple-pore architectures. Furthermore, reverse degree based entropy measures are computed to quantify structural orderness and to explore isentropic configurations within the TP-COF family. Using these structural parameters, quantitative structure-property models are constructed for spectral energy, demonstrating strong predictive capability. In addition, the spectral diameter and HOMO–LUMO gap are analyzed using graph spectral techniques, providing insights into the spectral characteristics of TP-COFs.

## 1 Introduction

Covalent organic frameworks have emerged as highly promising materials over the past decades due to their remarkable crystallinity, extensive surface areas, and wide-ranging applications, including catalysis, sensing, energy storage, drug delivery, optoelectronic devices, separation processes, and gas adsorption and storage (Bai et al., 2016; Ding et al., 2016; Kandambeth et al., 2017; Liao et al., 2016; Liu et al., 2026). As an emerging class of crystalline porous organic materials, the properties and performance of COFs strongly depend on the characteristics of their pores, which are determined by the topological structures of the networks and their sizes (Carrington et al., 2022; Liang et al., 2020; Yusran et al., 2024). In particular, appropriate pore architectures and functionalization enable COFs to act as efficient adsorbents for the capture of hazardous metal ions, organic and biological pollutants, as well as greenhouse gases (Ghazi et al., 2018; Li et al., 2021; Xiao et al., 2021; Xin et al., 2021). Traditionally, COFs have been constructed through the principles of reticular chemistry, which enable the precise prediction of their structures based on the symmetry and geometry of the building blocks used for condensation reactions (Abuzeid et al., 2021; Yaghi, 2016; Yu et al., 2022). The design strategies for COFs have therefore focused mainly on combining building units with compatible symmetries. Using this approach, various COFs with tetragonal, hexagonal, or triangular pores have been designed and synthesized since the first two COFs were reported in 2005 (Chen et al., 2025; Côté et al., 2005; Gao et al., 2018; Geng et al., 2020; Jing et al., 2023). In these traditional COFs, there is usually only one kind of pore in a given framework, which limits their structural complexity and functional diversity.

**FIGURE 1:**
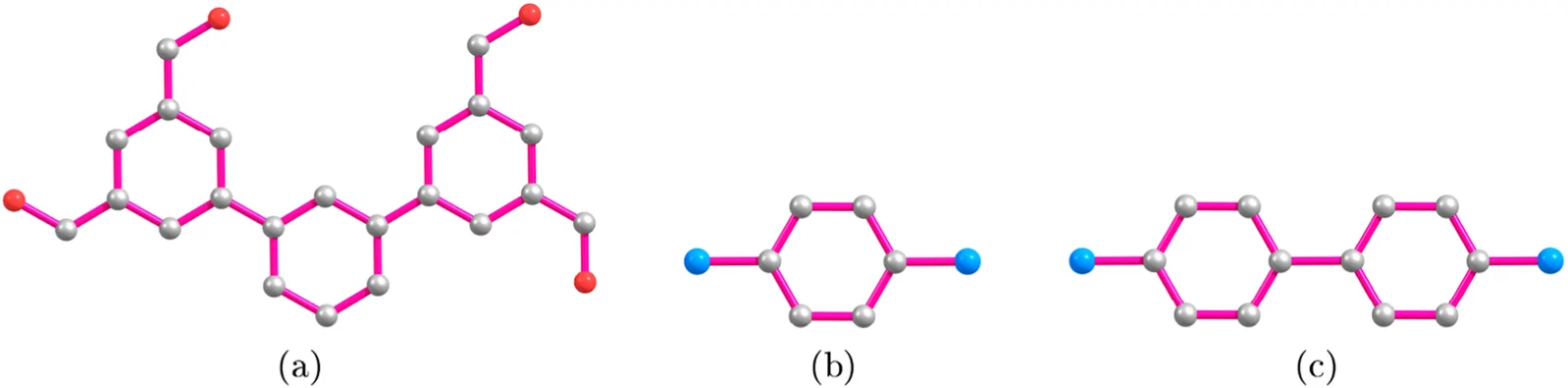
**(a)** $\left[1,1^{\prime }:3^{\prime },1^{\prime \prime }-\text{terphenyl}\right]-3,3^{\prime \prime },5,5^{\prime \prime }-\text{tetracarbaldehyde}$ **(b)** 1,4-diaminobenzene **(c)** benzidine.

**FIGURE 2:**
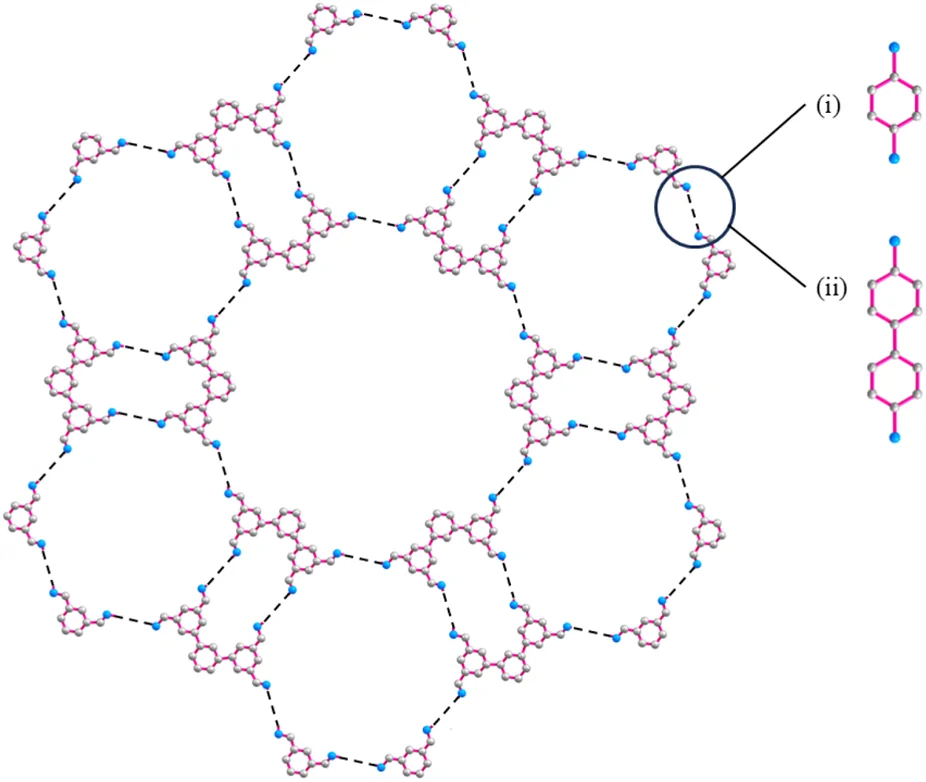
Structural units (i) TP-COF-DAB (ii) TP-COF-BZ.

Recent advances have introduced a paradigm shift with the development of heteropore COFs that incorporate two or three distinct types of pores within a single continuous network (Dalapati et al., 2016; Du et al., 2016; Han et al., 2020). These structures enrich the structural diversity of the COF family and open new avenues for multifunctionality. A pioneering design strategy combining vertex truncation and multiple linking site approaches has enabled the fabrication of triple-pore COFs (Qian et al., 2017). In this design, the building block $\left[1,1^{\prime }:3^{\prime },1^{\prime \prime }-\text{terphenyl}\right]-3,3^{\prime \prime },5,5^{\prime \prime }-\text{tetracarbaldehyde}$ (TPTCA) is shown in Figure 1a, while the linear linkers 1,4-diaminobenzene (DAB) and benzidine (BZ) are shown in Figures 1b,c. Condensation of TPTCA with these linkers yields the structural units for TP-COF-DAB and TP-COF-BZ, presented in Figure 2. These COFs exhibit an fxt topology, where the V-shaped geometry of the TPTCA unit is crucial for generating the connectivity that gives rise to three distinct pore types (Bhambri et al., 2022). This advancement reflects a broader challenge in polymer and materials science, moving beyond primary connectivity toward precisely controlled higher-order structures. While biological polymers achieve remarkable hierarchical organization, replicating such structural precision in synthetic systems remains a significant challenge (Kricheldorf, 2006). The design of multi-pore COFs such as TP-COF-DAB and TP-COF-BZ represents a meaningful step toward this goal, embodying the idea of tailor-made frameworks in materials design. Analyzing their intricate architectures is therefore not only of theoretical interest but also essential for understanding and exploiting their full functional potential.

Since the pore architecture is the primary factor governing the physical and chemical properties of COFs, a topological analysis that quantitatively characterizes the pore environment is essential. Topological indices, which are numerical descriptors derived from graph theory, have been widely employed to characterize the structure of molecular and extended frameworks (Arockiaraj et al., 2025a; Junias and Clement, 2024; Kalaam et al., 2024; Mondal et al., 2026; Tang et al., 2025). In the context of COFs, several studies have applied degree-based and related topological indices to correlate structural features with properties (Arockiaraj et al., 2025b; Augustine and Roy, 2022; Kurian et al., 2025; Tu et al., 2025; Zhang X. et al., 2025). These indices provide a powerful way to understand how the structure of the framework influences its physicochemical properties (Arockiaraj et al., 2025c; Manuel and Angamuthu, 2025; Paul et al., 2025; Raza et al., 2024). However, conventional degree-based topological indices mainly describe atom connectivity, which may not fully capture the complexity of the pores themselves. To address this limitation, a face-degree-based topological framework is employed, in which indices are defined in terms of face degrees, thereby providing a more direct and accurate representation of the pore structure. Notably, scaled face reverse-degree based indices have been shown to be effective for benzenoid hydrocarbons compared with conventional degree-based indices (Arockiaraj et al., 2026a). In parallel, reverse-degree topological indices offer an alternative perspective for capturing structural information by emphasizing complementary connectivity patterns within the network (Ahmad et al., 2023; Kalaam and Greeni, 2024; Kalaam and Greeni, 2025; Rao et al., 2024; Youssef et al., 2024). Consequently, the reverse-degree framework provides additional structural information that can be used for subsequent QSPR modeling (Aq et al., 2026; Ravi, 2024). Thus, in this study, we analyze TP-COFs with reverse-degree indices, and scaled face reverse-degree indices. We further investigate the existence of isentropic structures by means of reverse-degree based graph entropies, which have attracted considerable attention in the literature as measures of structural complexity and information content (Kavitha et al., 2021; Naeem et al., 2024; Zhang G. et al., 2025). In addition, entropy measures are relevant to both thermodynamics and information theory, which provide valuable insights into the behaviour of complex systems across scientific and engineering domains (Abraham et al., 2022; Hussain et al., 2025; Jawahar and Clement, 2026; Peter et al., 2025; Yu et al., 2024). Building upon these analyses, we further investigate spectral descriptors and develop predictive models for spectral graph energy using the computed topological indices.

## 2 Computational techniques

The triple-pore covalent organic frameworks investigated in this study are modeled as hydrogen-suppressed graph structures TP-COF=(V(TP-COF),E(TP-COF)) , where V(TP-COF) represents the set of vertices corresponding to atoms, and E(TP-COF) represents the set of edges corresponding to bonds. In this work, we aim to explore the degree parameter $d_{\text{TP}-\text{COF}}\left(x\right)$ of each vertex x∈V(TP-COF) , defined as the number of edges incident on the vertex, to its full potential. Accordingly, a modified reverse-degree parameter is employed to reveal different degree combinations and to represent vertices with lower connectivity. The reverse degree is defined as

$$r_{k}\left(x\right)=\left\{\begin{array}{cc} \Delta \left(\text{TP}-\text{COF}\right)-d_{\text{TP}-\text{COF}}\left(x\right)+k & :k\leq d_{\text{TP}-\text{COF}}\left(x\right)\\ \left(\Delta \left(\text{TP}-\text{COF}\right)-d_{\text{TP}-\text{COF}}\left(x\right)+k\right)\;\mathrm{mod}\Delta \left(\text{TP}-\text{COF}\right) & :k>d_{\text{TP}-\text{COF}}\left(x\right) \end{array}\right.$$ (1)

Here, Δ(TP-COF) denotes the maximum degree in TP-COF , and the reversing parameter k takes integer values from 1 to Δ(TP-COF) . For k>Δ(TP-COF) , the degree values repeat cyclically. The reverse-degree parameter defined in Equation 1 follows existing formulations in the literature, where reverse-degree concepts are employed to capture complementary connectivity patterns in complex networks. Such a definition has been effectively used in recent studies to improve degree-based indices (Ahmad et al., 2023; Kalaam and Greeni, 2024; Kalaam and Greeni, 2025; Rao et al., 2024; Youssef et al., 2024). In this work, the same formulation is adopted in the context of TP-COFs to appropriately reflect their connectivity variations. The degree-based topological indices are defined as $TI^{d}\left(\text{TP}-\text{COF}\right)=\sum_{xy\in E\left(\text{TP}-\text{COF}\right)}TI\left(d_{\text{TP}-\text{COF}}\left(x\right),d_{\text{TP}-\text{COF}}\left(y\right)\right)$ . In order to incorporate the reverse-degree parameters, we define the associated topological indices as

$$TI^{r_{k}}\left(\text{TP}-\text{COF}\right)=\underset{xy\in E\left(\text{TP}-\text{COF}\right)}{\sum }TI\left(r_{k}\left(x\right),r_{k}\left(y\right)\right)$$ (2)

Furthermore, to integrate the effect of reverse-degree modification with the structural influence of pores, we employ the face-reverse-degree indices for each face F∈F(TP-COF) , defined as

$$FTI^{r_{k}}\left(F\right)=\underset{xy\in E\left(F\right)}{\sum }TI\;\left(r_{k}\left(x\right),r_{k}\left(y\right)\right)$$ (3)

where E(F) denotes the set of boundary edges of the face F , and F(TP-COF) represents the set of all faces of TP-COF . The set F(TP-COF) includes both internal faces $\left(F_{i}\right)$ , which are completely bounded cycles, and the external face $\left(F_{e}\right)$ , which outlines the outer boundary of the structure. During the boundary traversal of a face, pendant edges are counted twice (Arockiaraj et al., 2026b). The total number of faces is given by the cardinality |F(TP-COF)| . Aggregating the face contributions derived from Equation 3, the scaled face-reverse-degree index of TP-COF is defined as

$$F^{*}TI^{r_{k}}\left(\text{TP}-\text{COF}\right)=\frac{\left|E\left(\text{TP}-\text{COF}\right)\right|}{\left|F\left(\text{TP}-\text{COF}\right)\right|}\underset{F\in F\left(\text{TP}-\text{COF}\right)}{\sum }\frac{FTI^{r_{k}}\left(F\right)}{\left|E\left(F\right)\right|}$$ (4)

To emphasize the structural influence of these reverse-degree modifications and their pore-level extension, we consider the first Zagreb $M_{1}\left(a,z\right)$ , second Zagreb $M_{2}\left(a,z\right)$ , hyper-Zagreb HM(a,z) , forgotten F(a,z) , arithmetic A(a,z) , bi-Zagreb BM(a,z) , tri-Zagreb TM(a,z) , bi-Zagreb-arithmetic BMA(a,z) , tri-Zagreb-harmonic TMH(a,z) , and tri-Zagreb-arithmetic TMA(a,z) , which are defined respectively as

$$\begin{array}{ll} TI\left(a,z\right)=\left\{\;\right. & a+z,\;az,\;{\left(a+z\right)}^{2},\;a^{2}+z^{2},\;\frac{\left(a+z\right)}{2},\;a+z+az,\;a^{2}+z^{2}+az,\;\\ & \left.\;\frac{2\left(a+z+az\right)}{a+z},\frac{\left(a^{2}+z^{2}+az\right)\left(a+z\right)}{2},\;\frac{2\left(a^{2}+z^{2}+az\right)}{a+z}\;\right\} \end{array}$$

Among these topological indices, the first Zagreb index was originally introduced in relation to the total π -electron energy of molecular graphs, while the forgotten index was later shown to provide improved structural sensitivity in subsequent investigations (Furtula and Gutman, 2015; Gutman and Trinajstić, 1972). In recent studies, these indices have been extended through reverse-degree and scaled face-based formulations to better represent structural variations (Arockiaraj et al., 2026a; Kalaam and Greeni, 2025). These extensions have been shown to provide additional structural information compared to existing indices and support their applicability in structural analysis and potential QSPR studies (Ahmad et al., 2023; Aq et al., 2026; Ravi, 2024).

## 3 Results and discussion

**FIGURE 3:**
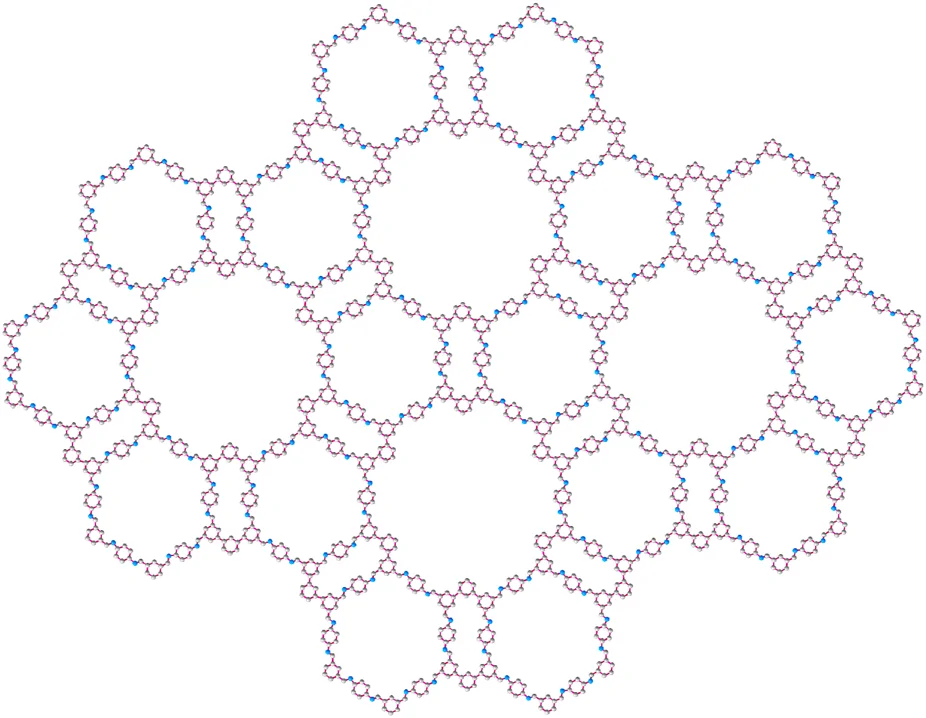
Bi-trapezium configuration of DAB(2,2) .

**FIGURE 4:**
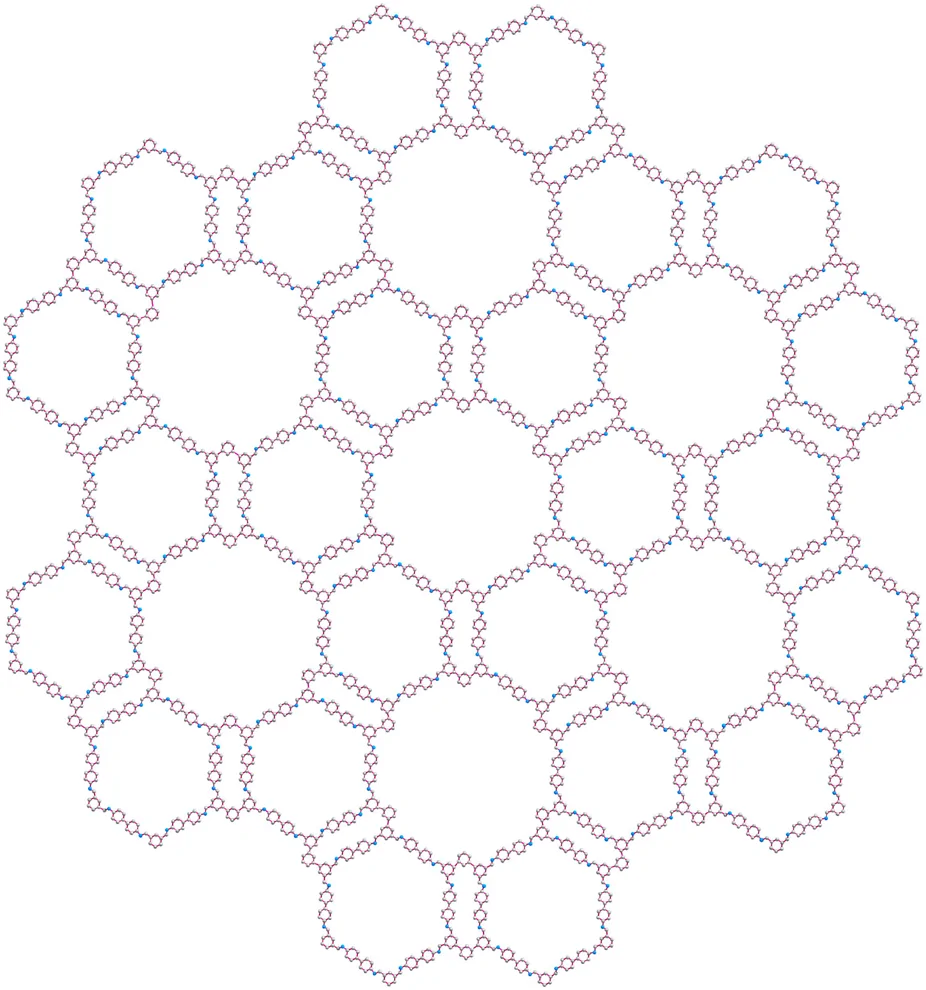
Bi-trapezium configuration of BZ(3,2) .

In TP-COFs, the structural units can be arranged in various configurations, such as linear, rectangular, hexagonal, parallelogram, and bi-trapezium, depending on the topology and connectivity of the building units. These configurations influence the overall geometry, porosity, and functional characteristics of the materials. In the present study, the bi-trapezium configuration is used to model the arrangement of structural units as graph structures. The representative bi-trapezium configurations considered in this work are illustrated in Figures 3, 4. These figures depict the structural arrangement of the TP-COFs, where vertices represent atoms and edges represent bonds. In particular, the figures highlight variations in connectivity and the arrangement of faces, which form the basis for computing the reverse-degree and scaled face reverse-degree topological indices. This configuration includes two important structural forms, namely linear and hexagonal, as subcases, and therefore provides a general framework suitable for representing a wide range of geometric variations observed in TP-COFs. Let DAB(n,m) and BZ(n,m) represent the bi-trapezium graph structures corresponding to the two TP-COFs, namely TP-COF-DAB and TP-COF-BZ, respectively. Here, n and m denote the structural parameters that determine the extent of the bi-trapezium arrangement. The vertex and edge set counts of these COFs are given, respectively, by $\left\{\left|V\left(DAB\right)|,|E\left(DAB\right)\right|\right\}=\{444m-12n+456mn-228m^{2}-12,512m$ $\;-16n+528mn-264m^{2}-16\}$ and $\left\{\left|V\left(BZ\right)|,|E\left(BZ\right)\right|\right\}=\{588m-12n+600mn-300m^{2}-12,680m$ $\;-16n+696mn-348m^{2}-16\}$ .

### 3.1 Reverse degree topological indices

To streamline the computation of the reverse-degree topological indices, we first partition the bonds into bond classes using the bond-partition technique, which groups edges according to the degrees of their end vertices. Mathematically, this is defined as

$$d\left(a,z\right)=\left|\left\{\;xy\in E\left(\text{TP}-\text{COF}\right)|d_{\text{TP}-\text{COF}}\left(x\right)=a,\;d_{\text{TP}-\text{COF}}\left(y\right)=z\;\right\}\right|.$$

Let D(TP-COF) denote the set of bond classes of the TP-COFs. Then, the frameworks DAB and BZ share the same bond classes, D(DAB)=D(BZ)={(2,2),(2,3),(3,3)} . However, their cardinalities differ, and the corresponding values are presented in Table 1.

**TABLE 1:** Bond partitions of DAB and BZ .

Bond classes	DAB	BZ
d(2,2)	$124m+4n+120mn-60m^{2}+4$	$172m+4n+168mn-84m^{2}+4$
d(2,3)	$368m-16n+384mn-192m^{2}-16$	$464m-16n+480mn-240m^{2}-16$
d(3,3)	$20m-4n+24mn-12m^{2}-4$	$44m-4n+48mn-24m^{2}-4$

From Table 1, the degree set of TP-COF is {2,3} , with maximum degree 3. Accordingly, the reversing parameter k ranges from 1 to 3, and the corresponding modifications are

$$d_{\text{TP}-\text{COF}}\left(x\right)=2,r_{k}\left(x\right)=\left\{\begin{array}{cc} \;2 & \;:k=1\\ \;3 & \;:k=2\\ \;1 & \;:k=3 \end{array}\right.$$

$$d_{\text{TP}-\text{COF}}\left(x\right)=3,r_{k}\left(x\right)=\left\{\begin{array}{cc} \;1 & \;:k=1\\ \;2 & \;:k=2\\ \;3 & \;:k=3 \end{array}\right.$$

Thus, the modified bond partitions for k=1 are given by $D^{r_{1}}\left(DAB\right)=D^{r_{1}}\left(BZ\right)=\left\{\left(2,2\right),\;\left(2,1\right),\;\left(1,1\right)\right\}$ . For k=2 , the partitions are $D^{r_{2}}\left(DAB\right)=D^{r_{2}}\left(BZ\right)=\left\{\left(3,3\right),\;\left(3,2\right),\;\left(2,2\right)\right\}$ and for k=3 , $D^{r_{3}}\left(DAB\right)=D^{r_{3}}\left(BZ\right)=\left\{\left(1,1\right),\;\left(1,3\right),\;\left(3,3\right)\right\}$ . The corresponding cardinalities remain unchanged as per order given in Table 1. Then, the general formula for reverse-degree topological indices, obtained by substituting into Equation 2, is given by

$$\begin{array}{cc} TI^{r_{k}}\left(DAB\right) & =\left(124m+4n+120mn-60m^{2}+4\right)\;TI\left(r_{k}\left(2\right),r_{k}\left(2\right)\right)\\ & +\left(368m-16n+384mn-192m^{2}-16\right)\;TI\left(r_{k}\left(2\right),r_{k}\left(3\right)\right)\\ & +\left(20m-4n+24mn-12m^{2}-4\right)\;TI\left(r_{k}\left(3\right),r_{k}\left(3\right)\right) \end{array}$$ (5)

$$\begin{array}{cc} TI^{r_{k}}\left(BZ\right) & =\left(172m+4n+168mn-84m^{2}+4\right)\;TI\left(r_{k}\left(2\right),r_{k}\left(2\right)\right)\\ & +\left(464m-16n+480mn-240m^{2}-16\right)\;TI\left(r_{k}\left(2\right),r_{k}\left(3\right)\right)\\ & +\left(44m-4n+48mn-24m^{2}-4\right)\;TI\left(r_{k}\left(3\right),r_{k}\left(3\right)\right) \end{array}$$ (6)

By substituting the topological indices into Equations 5 and 6, the corresponding closed-form expressions for the reverse-degree topological indices are obtained, and the results are presented in the form $TI^{r_{k}}=\left\{TI^{r_{1}},TI^{r_{2}},TI^{r_{3}}\right\}$ in Results 1 and 2.

Result 1 *For the bi-trapezium configuration* DAB *of dimension* (n,m) *, the reverse-degree indices are given by*

1.
$M_{1}^{r_{k}}\left(DAB\right)=\left\{1640\;m-40\;n+1680\;m\;n-840\;m^{2}-40,2664\;m-72\;n+2736\;m\;n-1368\;m^{2}-72,1840\;m-80\;n+1920\;m\;n-960\;m^{2}-80\right\}$

2.
$M_{2}^{r_{k}}\left(DAB\right)=\left\{1252\;m-20\;n+1272\;m\;n-636\;m^{2}-20,3404\;m-76\;n+3480\;m\;n-1740\;m^{2}-76,1408\;m-80\;n+1488\;m\;n-744\;m^{2}-80\right\}$

3.
$HM^{r_{k}}\left(DAB\right)=\left\{5376\;m-96\;n+5472\;m\;n-2736\;m^{2}-96,13984\;m-320\;n+14304\;m\;n-7152\;m^{2}-320,7104\;m-384\;n+7488\;m\;n-3744\;m^{2}-384\right\}$

4.
$F^{r_{k}}\left(DAB\right)=\left\{2872\;m-56\;n+2928\;m\;n-1464\;m^{2}-56,7176\;m-168\;n+7344\;m\;n-3672\;m^{2}-168,4288\;m-224\;n+4512\;m\;n-2256\;m^{2}-224\right\}$

5.
$A^{r_{k}}\left(DAB\right)=\left\{820\;m-20\;n+840\;m\;n-420\;m^{2}-20,1332\;m-36\;n+1368\;m\;n-684\;m^{2}-36,920\;m-40\;n+960\;m\;n-480\;m^{2}-40\right\}$

6.
$BM^{r_{k}}\left(DAB\right)=\left\{2892\;m-60\;n+2952\;m\;n-1476\;m^{2}-60,6068\;m-148\;n+6216\;m\;n-3108\;m^{2}-148,3248\;m-160\;n+3408\;m\;n-1704\;m^{2}-160\right\}$

7.
$TM^{r_{k}}\left(DAB\right)=\left\{4124\;m-76\;n+4200\;m\;n-2100\;m^{2}-76,10580\;m-244\;n+10824\;m\;n-5412\;m^{2}-244,5696\;m-304\;n+6000\;m\;n-3000\;m^{2}-304\right\}$

8.
$BMA^{r_{k}}\left(DAB\right)=\left\{\left(5348\;m-148\;n+5496\;m\;n-2748\;m^{2}-148\right)/3,\left(11596\;m-332\;n+11928\;m\;n-5964\;m^{2}-332\right)/5,1760\;m-64\;n+1824\;m\;n-912\;m^{2}-64\right\}$

9.
$TMH^{r_{k}}\left(DAB\right)=\left\{6900\;m-84\;n+6984\;m\;n-3492\;m^{2}-84,28004\;m-532\;n+28536\;m\;n-14268\;m^{2}-532,11560\;m-728\;n+12288\;m\;n-6144\;m^{2}-728\right\}$

10.
$TMA^{r_{k}}\left(DAB\right)=\left\{\left(7564\;m-188\;n+7752\;m\;n-3876\;m^{2}-188\right)/3,\left(20164\;m-548\;n+20712\;m\;n-10356\;m^{2}-548\right)/5,2944\;m-128\;n+3072\;m\;n-1536\;m^{2}-128\right\}$

Result 2 *For the bi-trapezium configuration* BZ *of dimension* (n,m) *, the reverse-degree indices are given by*

1.
$M_{1}^{r_{k}}\left(BZ\right)=\left\{2168\;m-40\;n+2208\;m\;n-1104\;m^{2}-40,3528\;m-72\;n+3600\;m\;n-1800\;m^{2}-72,2464\;m-80\;n+2544\;m\;n-1272\;m^{2}-80\right\}$

2.
$M_{2}^{r_{k}}\left(BZ\right)=\left\{1660\;m-20\;n+1680\;m\;n-840\;m^{2}-20,4508\;m-76\;n+4584\;m\;n-2292\;m^{2}-76,1960\;m-80\;n+\;2040\;m\;n-1020\;m^{2}-80\right\}$

3.
$HM^{r_{k}}\left(BZ\right)=\left\{7104\;m-96\;n+7200\;m\;n-3600\;m^{2}-96,18496\;m-320\;n+18816\;m\;n-9408\;m^{2}-320,9696\;m-384\;n+\;10080\;m\;n-5040\;m^{2}-384\right\}$

4.
$F^{r_{k}}\left(BZ\right)=\left\{3784\;m-56\;n+3840\;m\;n-1920\;m^{2}-56,9480\;m-168\;n+9648\;m\;n-4824\;m^{2}-168,5776\;m-224\;n+\;6000\;m\;n-3000\;m^{2}-224\right\}$

5.
$A^{r_{k}}\left(BZ\right)=\left\{1084\;m-20\;n+1104\;m\;n-552\;m^{2}-20,1764\;m-36\;n+1800\;m\;n-900\;m^{2}-36,1232\;m-40\;n+\;1272\;m\;n-636\;m^{2}-40\right\}$

6.
$BM^{r_{k}}\left(BZ\right)=\left\{3828\;m-60\;n+3888\;m\;n-1944\;m^{2}-60,8036\;m-148\;n+8184\;m\;n-4092\;m^{2}-148,4424\;m-160\;n+\;4584\;m\;n-2292\;m^{2}-160\right\}$

7.
$TM^{r_{k}}\left(BZ\right)=\left\{5444\;m-76\;n+5520\;m\;n-2760\;m^{2}-76,13988\;m-244\;n+14232\;m\;n-7116\;m^{2}-244,7736\;m-304\;n+8040\;m\;n-\;4020\;m^{2}-304\right\}$

8.
$BMA^{r_{k}}\left(BZ\right)=\left\{\left(7100\;m-148\;n+7248\;m\;n-3624\;m^{2}-148\right)/3,\left(15388\;m-332\;n+15720\;m\;n-7860\;m^{2}-332\right)/5,2360\;m-\;64\;n+2424\;m\;n-1212\;m^{2}-64\right\}$

9.
$TMH^{r_{k}}\left(BZ\right)=\left\{9132\;m-84\;n+9216\;m\;n-4608\;m^{2}-84,37028\;m-532\;n+37560\;m\;n-18780\;m^{2}-532,16144\;m-\;728\;n+16872\;m\;n-8436\;m^{2}-728\right\}$

10.
$TMA^{r_{k}}\left(BZ\right)=\left\{\left.9988\;m-188\;n+10176\;m\;n-5088\;m^{2}-188\right)/3,\left(26692\;m-548\;n+27240\;m\;n-13620\;m^{2}-548\right)/5,3928\;m-\;128\;n+4056\;m\;n-2028\;m^{2}-128\right\}$

Based on Results 1–2, the reverse-degree topological indices of DAB and BZ for k=1,2,3 are computed, and the values are presented in Tables 2–4.

**TABLE 2:** Reverse-degree (k=1) topological indices of DAB and BZ for varying (n,m) .

$TI^{r_{1}}$	$M_{1}^{r_{1}}$	$M_{2}^{r_{1}}$	$HM^{r_{1}}$	$F^{r_{1}}$	$A^{r_{1}}$	$BM^{r_{1}}$	$TM^{r_{1}}$	$BMA^{r_{1}}$	$TMH^{r_{1}}$	$TMA^{r_{1}}$
DAB(1,1)	2400	1848	7920	4224	1200	4248	6072	2600	10224	3688
DAB(2,1)	4040	3100	13296	7096	2020	7140	10196	4382.6667	17124	6209.3333
DAB(2,2)	6520	4988	21408	11432	3260	11508	16420	7081.3333	27516	10022.6667
DAB(3,1)	5680	4352	18672	9968	2840	10032	14320	6165.3333	24024	8730.6667
DAB(3,2)	9840	7512	32256	17232	4920	17352	24744	10696	41400	15128
DAB(4,1)	7320	5604	24048	12840	3660	12924	18444	7948	30924	11252
DAB(4,2)	13160	10036	43104	23032	6580	23196	33068	14310.6667	55284	20233.3333
DAB(4,3)	17320	13196	56688	30296	8660	30516	43492	18841.3333	72660	26630.6667
DAB(5,1)	8960	6856	29424	15712	4480	15816	22568	9730.6667	37824	13773.3333
DAB(5,2)	16480	12560	53952	28832	8240	29040	41392	17925.3333	69168	25338.6667
DAB(5,3)	22320	16992	73008	39024	11160	39312	56016	24288	93528	34320
DAB(5,4)	26480	20152	86592	46288	13240	46632	66440	28818.6667	110904	40717.3333
BZ(1,1)	3192	2460	10512	5592	1596	5652	8052	3476	13572	4900
BZ(2,1)	5360	4120	17616	9376	2680	9480	13496	5842.6667	22704	8229.3333
BZ(2,2)	8632	6620	28320	15080	4316	15252	21700	9417.3333	36444	13254.6667
BZ(3,1)	7528	5780	24720	13160	3764	13308	18940	8209.3333	31836	11558.6667
BZ(3,2)	13008	9960	42624	22704	6504	22968	32664	14200	54792	19976
BZ(4,1)	9696	7440	31824	16944	4848	17136	24384	10576	40968	14888
BZ(4,2)	17384	13300	56928	30328	8692	30684	43628	18982.6667	73140	26697.3333
BZ(4,3)	22864	17480	74832	39872	11432	40344	57352	24973.3333	96096	35114.6667
BZ(5,1)	11864	9100	38928	20728	5932	20964	29828	12942.6667	50100	18217.3333
BZ(5,2)	21760	16640	71232	37952	10880	38400	54592	23765.3333	91488	33418.6667
BZ(5,3)	29448	22500	96336	51336	14724	51948	73836	32172	123660	45228
BZ(5,4)	34928	26680	114240	60880	17464	61608	87560	38162.6667	146616	53645.3333

**TABLE 3:** Reverse-degree (k=2) topological indices of DAB and BZ for varying (n,m) .

$TI^{r_{2}}$	$M_{1}^{r_{2}}$	$M_{2}^{r_{2}}$	$HM^{r_{2}}$	$F^{r_{2}}$	$A^{r_{2}}$	$BM^{r_{2}}$	$TM^{r_{2}}$	$BMA^{r_{2}}$	$TMH^{r_{2}}$	$TMA^{r_{2}}$
DAB(1,1)	3888	4992	20496	10512	1944	8880	15504	3379.2	41208	5884.8
DAB(2,1)	6552	8396	34480	17688	3276	14948	26084	5698.4	69212	9917.6
DAB(2,2)	10584	13540	55616	28536	5292	24124	42076	9210.4	111484	16021.6
DAB(3,1)	9216	11800	48464	24864	4608	21016	36664	8017.6	97216	13950.4
DAB(3,2)	15984	20424	83904	43056	7992	36408	63480	13915.2	168024	24196.8
DAB(4,1)	11880	15204	62448	32040	5940	27084	47244	10336.8	125220	17983.2
DAB(4,2)	21384	27308	112192	57576	10692	48692	84884	18620	224564	32372
DAB(4,3)	28152	35932	147632	75768	14076	64084	111700	24517.6	295372	42618.4
DAB(5,1)	14544	18608	76432	39216	7272	33152	57824	12656	153224	22016
DAB(5,2)	26784	34192	140480	72096	13392	60976	106288	23324.8	281104	40547.2
DAB(5,3)	36288	46296	190224	97632	18144	82584	143928	31608	380448	54936
DAB(5,4)	43056	54920	225664	115824	21528	97976	170744	37505.6	451256	65182.4
BZ(1,1)	5184	6648	27264	13968	2592	11832	20616	4516.8	54744	7843.2
BZ(2,1)	8712	11156	45760	23448	4356	19868	34604	7594.4	91772	13181.6
BZ(2,2)	14040	17956	73664	37752	7020	31996	55708	12244	147580	21244
BZ(3,1)	12240	15664	64256	32928	6120	27904	48592	10672	128800	18520
BZ(3,2)	21168	27048	110976	56880	10584	48216	83928	18465.6	222168	32030.4
BZ(4,1)	15768	20172	82752	42408	7884	35940	62580	13749.6	165828	23858.4
BZ(4,2)	28296	36140	148288	76008	14148	64436	112148	24687.2	296756	42816.8
BZ(4,3)	37224	47524	195008	99960	18612	84748	147484	32480.8	390124	56327.2
BZ(5,1)	19296	24680	101248	51888	9648	43976	76568	16827.2	202856	29196.8
BZ(5,2)	35424	45232	185600	95136	17712	80656	140368	30908.8	371344	53603.2
BZ(5,3)	47952	61200	251136	128736	23976	109152	189936	41846.4	502272	72561.6
BZ(5,4)	56880	72584	297856	152688	28440	129464	225272	49640	595640	86072

**TABLE 4:** Reverse-degree (k=3) topological indices of DAB and BZ for varying (n,m) .

$TI^{r_{3}}$	$M_{1}^{r_{3}}$	$M_{2}^{r_{3}}$	$HM^{r_{3}}$	$F^{r_{3}}$	$A^{r_{3}}$	$BM^{r_{3}}$	$TM^{r_{3}}$	$BMA^{r_{3}}$	$TMH^{r_{3}}$	$TMA^{r_{3}}$
DAB(1,1)	2640	1992	10080	6096	1320	4632	8088	2544	16248	4224
DAB(2,1)	4480	3400	17184	10384	2240	7880	13784	4304	27808	7168
DAB(2,2)	7280	5552	28032	16928	3640	12832	22480	6976	45512	11648
DAB(3,1)	6320	4808	24288	14672	3160	11128	19480	6064	39368	10112
DAB(3,2)	11040	8448	42624	25728	5520	19488	34176	10560	69360	17664
DAB(4,1)	8160	6216	31392	18960	4080	14376	25176	7824	50928	13056
DAB(4,2)	14800	11344	57216	34528	7400	26144	45872	14144	93208	23680
DAB(4,3)	19520	14984	75552	45584	9760	34504	60568	18640	123200	31232
DAB(5,1)	10000	7624	38496	23248	5000	17624	30872	9584	62488	16000
DAB(5,2)	18560	14240	71808	43328	9280	32800	57568	17728	117056	29696
DAB(5,3)	25200	19368	97632	58896	12600	44568	78264	24048	159336	40320
DAB(5,4)	29920	23008	115968	69952	14960	52928	92960	28544	189328	47872
BZ(1,1)	3576	2820	13968	8328	1788	6396	11148	3444	23124	5700
BZ(2,1)	6040	4780	23664	14104	3020	10820	18884	5804	39268	9628
BZ(2,2)	9776	7760	38400	22880	4888	17536	30640	9376	63848	15584
BZ(3,1)	8504	6740	33360	19880	4252	15244	26620	8164	55412	13556
BZ(3,2)	14784	11760	58176	34656	7392	26544	46416	14160	96864	23568
BZ(4,1)	10968	8700	43056	25656	5484	19668	34356	10524	71556	17484
BZ(4,2)	19792	15760	77952	46432	9896	35552	62192	18944	129880	31552
BZ(4,3)	26072	20780	102768	61208	13036	46852	81988	24940	171332	41564
BZ(5,1)	13432	10660	52752	31432	6716	24092	42092	12884	87700	21412
BZ(5,2)	24800	19760	97728	58208	12400	44560	77968	23728	162896	39536
BZ(5,3)	33624	26820	132624	78984	16812	60444	105804	32148	221220	53604
BZ(5,4)	39904	31840	157440	93760	19952	71744	125600	38144	262672	63616

### 3.2 Scaled face reverse degree topological indices

**FIGURE 5:**
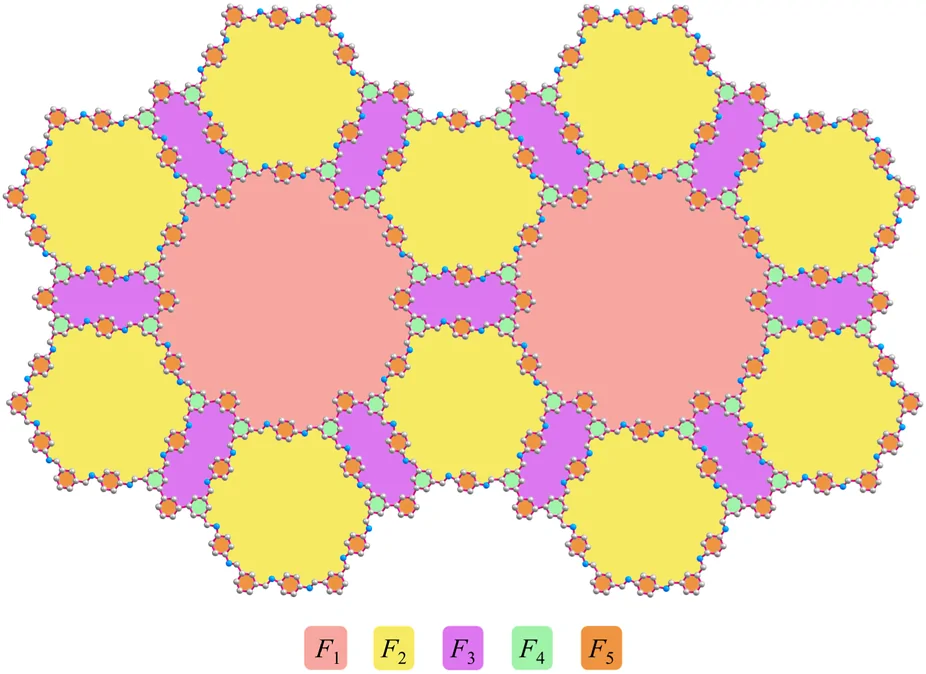
Graphical representation of the internal face partitions of DAB .

**FIGURE 6:**
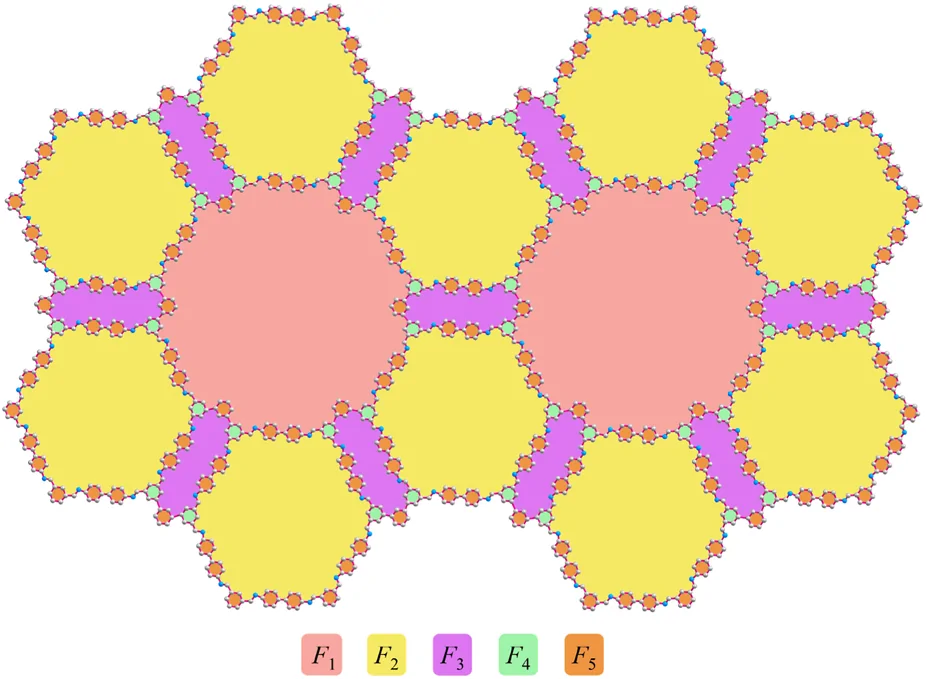
Graphical representation of the internal face partitions of BZ .

In the case of scaled face reverse degree topological indices, the TP-COFs are partitioned into faces. For both DAB and BZ , the face partitions consist of five internal faces $\left(F_{i}\right)$ and one external face $\left(F_{e}\right)$ , where the internal face partitions are illustrated in Figures 5, 6. In particular, these figures illustrate the classification of faces using different colours, where each colour corresponds to a distinct face class. Higher-dimensional structures follow a similar pattern. The cardinalities of the faces $F_{1},F_{2},F_{3},F_{4}$ , and $F_{e}$ are the same for both TP-COFs. However, the edge composition of each face, represented by $n_{\text{TP}-\text{COF}}^{F_{i}}\left(t,j\right)$ , varies. An exception occurs for the face $F_{5}$ , whose cardinality varies while its edge composition remains the same. The corresponding cardinalities and edge compositions are presented in Table 5. Moreover, the total number of faces in DAB and BZ are given by $68m-4n+72mn-36m^{2}-2$ and $92m-4n+96mn-48m^{2}-2$ , respectively. Let $\alpha_{\text{TP}-\text{COF}}=\frac{\left|E\left(\text{TP}-\text{COF}\right)\right|}{\left|F\left(\text{TP}-\text{COF}\right)\right|}$ . Then $\alpha_{DAB}=\frac{512m-16n+528mn-264m^{2}-16}{68m-4n+72mn-36m^{2}-2}$ and $\alpha_{BZ}=\frac{680m-16n+696mn-348m^{2}-16}{92m-4n+96mn-48m^{2}-2}$ . On substituting $\alpha_{\text{TP}-\text{COF}}$ and the modified reverse-degrees into Equation 4, the general formulas for scaled face reverse-degree topological indices are presented below.

$$\begin{array}{cc} F^{*}TI\left(DAB\right)=\alpha_{DAB}\left[\;\right. & \;\frac{\left|F_{1}\right|}{114}\left(30\;TI\left(r_{k}\left(2\right),r_{k}\left(2\right)\right)+12\;TI\left(r_{k}\left(3\right),r_{k}\left(3\right)\right)+72\;TI\left(r_{k}\left(2\right),r_{k}\left(3\right)\right)\right)\\ & +\frac{\left|F_{2}\right|}{66}\left(18\;TI\left(r_{k}\left(2\right),r_{k}\left(2\right)\right)+48\;TI\left(r_{k}\left(2\right),r_{k}\left(3\right)\right)\right)\\ & +\frac{\left|F_{3}\right|}{34}\left(6\;TI\left(r_{k}\left(2\right),r_{k}\left(2\right)\right)+4\;TI\left(r_{k}\left(3\right),r_{k}\left(3\right)\right)+24\;TI\left(r_{k}\left(2\right),r_{k}\left(3\right)\right)\right)\\ & +\frac{\left|F_{4}\right|}{6}\left(6\;TI\left(r_{k}\left(2\right),r_{k}\left(3\right)\right)\right)+\frac{\left|F_{5}\right|}{6}\left(2\;TI\left(r_{k}\left(2\right),r_{k}\left(2\right)\right)+4\;TI\left(r_{k}\left(2\right),r_{k}\left(3\right)\right)\right)\\ & +\frac{\left|F_{e}\right|}{128m+128n+14}\left(\left(40m+40n+10\right)\;TI\left(r_{k}\left(2\right),r_{k}\left(2\right)\right)\right.\\ & +\left.\left.\left(8m+8n-4\right)\;TI\left(r_{k}\left(3\right),r_{k}\left(3\right)\right)+\left(80m+80n+8\right)\;TI\left(r_{k}\left(2\right),r_{k}\left(3\right)\right)\right)\;\right] \end{array}$$ (7)

$$\begin{array}{cc} F^{*}TI\left(BZ\right)=\alpha_{BZ}\left[\;\right. & \;\frac{\left|F_{1}\right|}{138}\left(36\;TI\left(r_{k}\left(2\right),r_{k}\left(2\right)\right)+18\;TI\left(r_{k}\left(3\right),r_{k}\left(3\right)\right)+84\;TI\left(r_{k}\left(2\right),r_{k}\left(3\right)\right)\right)\\ & +\frac{\left|F_{2}\right|}{90}\left(24\;TI\left(r_{k}\left(2\right),r_{k}\left(2\right)\right)+6\;TI\left(r_{k}\left(3\right),r_{k}\left(3\right)\right)+60\;TI\left(r_{k}\left(2\right),r_{k}\left(3\right)\right)\right)\\ & +\frac{\left|F_{3}\right|}{42}\left(8\;TI\left(r_{k}\left(2\right),r_{k}\left(2\right)\right)+6\;TI\left(r_{k}\left(3\right),r_{k}\left(3\right)\right)+28\;TI\left(r_{k}\left(2\right),r_{k}\left(3\right)\right)\right)\\ & +\frac{\left|F_{4}\right|}{6}\left(6\;TI\left(r_{k}\left(2\right),r_{k}\left(3\right)\right)\right)+\frac{\left|F_{5}\right|}{6}\left(2\;TI\left(r_{k}\left(2\right),r_{k}\left(2\right)\right)+4\;TI\left(r_{k}\left(2\right),r_{k}\left(3\right)\right)\right)\\ & +\frac{\left|F_{e}\right|}{160m+160n+22}\left(\left(48m+48n+12\right)\;TI\left(r_{k}\left(2\right),r_{k}\left(2\right)\right)+\left(16m+16n-2\right)\;\right.\\ & \left.\left.TI\left(r_{k}\left(3\right),r_{k}\left(3\right)\right)+\left(96m+96n+12\right)\;TI\left(r_{k}\left(2\right),r_{k}\left(3\right)\right)\right)\;\right] \end{array}$$ (8)

**TABLE 5:** Cardinalities and edge compositions of the face partitions of DAB and BZ .

$F_{i}$	$\left|F_{i}\right|$	DAB	BZ
$F_{1}$	mn−m(m−1)+n(m−1)	$n_{DAB}^{F_{1}}\left(2,2\right)=30$	$n_{BZ}^{F_{1}}\left(2,2\right)=36$
		$n_{DAB}^{F_{1}}\left(3,3\right)=12$	$n_{BZ}^{F_{1}}\left(3,3\right)=18$
		$n_{DAB}^{F_{1}}\left(2,3\right)=72$	$n_{BZ}^{F_{1}}\left(2,3\right)=84$
$F_{2}$	2m(2n−m+2)	$n_{DAB}^{F_{2}}\left(2,2\right)=18$	$n_{BZ}^{F_{2}}\left(2,2\right)=24$
		$n_{DAB}^{F_{2}}\left(3,3\right)=0$	$n_{BZ}^{F_{1}}\left(3,3\right)=6$
		$n_{DAB}^{F_{2}}\left(2,3\right)=48$	$n_{BZ}^{F_{2}}\left(2,3\right)=60$
$F_{3}$	$5m-n+6mn-3m^{2}-1$	$n_{DAB}^{F_{3}}\left(2,2\right)=6$	$n_{BZ}^{F_{3}}\left(2,2\right)=8$
		$n_{DAB}^{F_{3}}\left(3,3\right)=4$	$n_{BZ}^{F_{3}}\left(3,3\right)=6$
		$n_{DAB}^{F_{3}}\left(2,3\right)=24$	$n_{BZ}^{F_{3}}\left(2,3\right)=28$
$F_{4}$	$20m-4n+24mn-12m^{2}-4$	$n_{DAB}^{F_{4}}\left(2,3\right)=6$	$n_{BZ}^{F_{4}}\left(2,3\right)=6$
$F_{5}\left(DAB\right)$	$38m+2n+36mn-18m^{2}+2$	$n_{DAB}^{F_{5}}\left(2,2\right)=2$	$n_{BZ}^{F_{5}}\left(2,2\right)=2$
$F_{5}\left(BZ\right)$	$62m+2n+60mn-30m^{2}+2$	$n_{DAB}^{F_{5}}\left(2,3\right)=4$	$n_{BZ}^{F_{5}}\left(2,3\right)=4$
$F_{e}$	1	$n_{DAB}^{F_{e}}\left(2,2\right)=40m+40n+10$	$n_{BZ}^{F_{e}}\left(2,2\right)=48m+48n+12$
		$n_{DAB}^{F_{e}}\left(3,3\right)=8m+8n-4$	$n_{BZ}^{F_{e}}\left(3,3\right)=16m+16n-2$
		$n_{DAB}^{F_{e}}\left(2,3\right)=80m+80n+8$	$n_{BZ}^{F_{e}}\left(2,3\right)=96m+96n+12$

By substituting the topological indices into Equations 7 and 8, the closed-form expressions for the scaled face reverse-degree-based topological indices are presented as $F^{*}TI^{r_{k}}=\left\{F^{*}TI^{r_{1}},F^{*}TI^{r_{2}},F^{*}TI^{r_{3}}\right\}$ in Results 3 and 4.

Result 3 *For the bi-trapezium configuration* DAB *of dimension* (n,m) *, the scaled face reverse-degree indices are given by*

1.
$F^{*}M_{1}^{r_{k}}\left(DAB\right)=\left\{\alpha_{DAB}\left(156736512\;n^{2}\;m-7879168\;n^{2}+78368256\;n\;m^{2}+158121232\;n\;m-4369640\;n-78368256\;m^{3}+140285816\;m^{2}+12773416\;m-327712\right)/\left(682176\;m+682176\;n+74613\right),\;\alpha_{DAB}\left(254969856\;n^{2}\;m-13336576\;n^{2}+127484928\;n\;m^{2}+256184032\;n\;m-7695248\;n-127484928\;m^{3}+227689616\;m^{2}+20192080\;m-626164\right)/\left(682176\;m+682176\;n+74613\right),\alpha_{DAB}\left(177693696\;n^{2}\;m-\;11528704\;n^{2}+88846848\;n\;m^{2}+174071536\;n\;m-7888760\;n-88846848\;m^{3}+156447368\;m^{2}+11546488\;m-836836\right)/\left(682176\;m+682176\;n+74613\right)\right\}$

2.
$F^{*}M_{2}^{r_{k}}\left(DAB\right)=\left\{\alpha_{DAB}\left(117631488\;n^{2}\;m-4995584\;n^{2}+58815744\;n\;m^{2}+120506264\;n\;m-2142316\;n-58815744\;m^{3}+106202932\;m^{2}+10723628\;m-88616\right)/\left(682176\;m+682176\;n+74613\right),\;\alpha_{DAB}\left(107828224\;n^{2}\;m-5201152\;n^{2}+53914112\;n\;m^{2}+109219632\;n\;m-2724920\;n-53914112\;m^{3}+96730216\;m^{2}+9068792\;m-188518\right)/\left(227392\;m+227392\;n+24871\right),\alpha_{DAB}\left(131077632\;n^{2}\;m-9408256\;n^{2}\;+65538816\;n\;m^{2}+126597736\;n\;m-6442964\;n-65538816\;m^{3}+114501068\;m^{2}+7893652\;m-807994\right)/\;\left(682176\;m+682176\;n+74613\right)\right\}$

3.
$F^{*}HM^{r_{k}}\left(DAB\right)=\left\{\alpha_{DAB}\left(509005824\;n^{2}\;m-22713856\;n^{2}+254502912\;n\;m^{2}+519250624\;n\;m-\;10742336\;n-254502912\;m^{3}+458455712\;m^{2}+44930176\;m-563464\right)/\left(682176\;m+682176\;n+74613\right),\;\alpha_{DAB}\left(121128960\;n^{2}\;m-5922304\;n^{2}+60564480\;n\;m^{2}+122532832\;n\;m-3170192\;n-60564480\;m^{3}+108582416\;m^{2}+10078288\;m-224656\right)/\left(62016\;m+62016\;n+6783\right),\alpha_{DAB}\left(226076672\;n^{2}\;m-16186368\;n^{2}\;+113038336\;n\;m^{2}+218431072\;n\;m-11488048\;n-113038336\;m^{3}+197526736\;m^{2}+13239088\;m-1355992\right)/\left(227392\;m+227392\;n+24871\right)\right\}$

4.
$F^{*}F^{r_{k}}\left(DAB\right)=\left\{\alpha_{DAB}\left(91247616\;n^{2}\;m-4240896\;n^{2}+45623808\;n\;m^{2}+92746032\;n\;m-2152568\;n-\;45623808\;m^{3}+82016616\;m^{2}+7827640\;m-128744\right)/\left(227392\;m+227392\;n+24871\right),\alpha_{DAB}\left(685449216\;n^{2}\;m-33938432\;n^{2}+342724608\;n\;m^{2}+692543360\;n\;m-18522592\;n-342724608\;m^{3}+614025280\;m^{2}+56448416\;m-1340108\right)/\left(682176\;m+682176\;n+74613\right),\alpha_{DAB}\left(416074752\;n^{2}\;m-29742592\;n^{2}+208037376\;n\;m^{2}+402097744\;n\;m-21578216\;n-208037376\;m^{3}+363578072\;m^{2}+23929960\;m-2451988\right)/\left(682176\;m+682176\;n+74613\right)\right\}$

5.
$F^{*}A^{r_{k}}\left(DAB\right)=\left\{\alpha_{DAB}\left(78368256\;n^{2}\;m-3939584\;n^{2}+39184128\;n\;m^{2}+79060616\;n\;m-2184820\;n-\;39184128\;m^{3}+70142908\;m^{2}+6386708\;m-163856\right)/\left(682176\;m+682176\;n+74613\right),\;\alpha_{DAB}\left(127484928\;n^{2}\;m-6668288\;n^{2}+63742464\;n\;m^{2}+128092016\;n\;m-3847624\;n-63742464\;m^{3}+113844808\;m^{2}+10096040\;m-313082\right)/\left(682176\;m+682176\;n+74613\right),\alpha_{DAB}\left(88846848\;n^{2}\;m-\;5764352\;n^{2}+44423424\;n\;m^{2}+87035768\;n\;m-3944380\;n-44423424\;m^{3}+78223684\;m^{2}+5773244\;m-418418\right)/\left(682176\;m+682176\;n+74613\right)\right\}$

6.
$F^{*}BM^{r_{k}}\left(DAB\right)=\left\{\alpha_{DAB}\left(91456000\;n^{2}\;m-4291584\;n^{2}+45728000\;n\;m^{2}+92875832\;n\;m-2170652\;n-45728000\;m^{3}+82162916\;m^{2}+7832348\;m-138776\right)/\left(227392\;m+227392\;n+24871\right),\;\alpha_{DAB}\left(578454528\;n^{2}\;m-28940032\;n^{2}+289227264\;n\;m^{2}+583842928\;n\;m-15870008\;n-289227264\;m^{3}+517880264\;m^{2}+47398456\;m-1191718\right)/\left(682176\;m+682176\;n+74613\right),\alpha_{DAB}\left(308771328\;n^{2}\;m-\;20936960\;n^{2}+154385664\;n\;m^{2}+300669272\;n\;m-14331724\;n-154385664\;m^{3}+270948436\;m^{2}+19440140\;m-1644830\right)/\left(682176\;m+682176\;n+74613\right)\right\}$

7.
$F^{*}TM^{r_{k}}\left(DAB\right)=\left\{\alpha_{DAB}\left(391374336\;n^{2}\;m-17718272\;n^{2}+195687168\;n\;m^{2}+398744360\;n\;m-\;8600020\;n-195687168\;m^{3}+352252780\;m^{2}+34206548\;m-474848\right)/\left(682176\;m+682176\;n+74613\right),\;\alpha_{DAB}\left(1008933888\;n^{2}\;m-49541888\;n^{2}+504466944\;n\;m^{2}+1020202256\;n\;m-26697352\;n-504466944\;m^{3}\;+904215928\;m^{2}+83654792\;m-1905662\right)/\left(682176\;m+682176\;n+74613\right),\alpha_{DAB}\left(547152384\;n^{2}\;m-\;39150848\;n^{2}+273576192\;n\;m^{2}\;+528695480\;n\;m-28021180\;n-273576192\;m^{3}+478079140\;m^{2}+31823612\;m-3259982\right)/\left(682176\;m+682176\;n+74613\right)\right\}$

8.
$F^{*}BMA^{r_{k}}\left(DAB\right)=\left\{\alpha_{DAB}\left(510564864\;n^{2}\;m-26825216\;n^{2}+255282432\;n\;m^{2}+512757464\;n\;m-15444748\;n-255282432\;m^{3}+455818132\;m^{2}+40398284\;m-1282424\right)/\left(2046528\;m+2046528\;n+223839\right),\alpha_{DAB}\left(369783808\;n^{2}\;m-19754240\;n^{2}+184891904\;n\;m^{2}+370720432\;n\;m-11593208\;n-184891904\;m^{3}+329807016\;m^{2}+28851896\;m-984390\right)/\left(1136960\;m+1136960\;n+124355\right),\;\alpha_{DAB}\left(167840256\;n^{2}\;m-9856000\;n^{2}+83920128\;n\;m^{2}+166485784\;n\;m-6183452\;n-83920128\;m^{3}+148805492\;m^{2}+12174076\;m-612370\right)/\left(682176\;m+682176\;n+74613\right)\right\}$

9.
$F^{*}TMH^{r_{k}}\left(DAB\right)=\left\{\alpha_{DAB}\left(12670464\;n^{2}\;m-498432\;n^{2}+6335232\;n\;m^{2}+13059432\;n\;m-184932\;n-\;6335232\;m^{3}+11479116\;m^{2}+1200900\;m-2508\right)/\left(13376\;m+13376\;n+1463\right),\alpha_{DAB}\left(2653741056\;n^{2}\;m-120851456\;n^{2}+1326870528\;n\;m^{2}+2702291072\;n\;m-58796848\;n-1326870528\;m^{3}+2387763136\;m^{2}+231456080\;m-3370334\right)/\left(682176\;m+682176\;n+74613\right),\alpha_{DAB}\left(1081148928\;n^{2}\;m-82872064\;n^{2}+\;540574464\;n\;m^{2}+1033655464\;n\;m-59200724\;n-540574464\;m^{3}+939151532\;m^{2}+59049940\;m-7602166\right)/\left(682176\;m+682176\;n+74613\right)\right\}$

10.
$F^{*}TMA^{r_{k}}\left(DAB\right)=\left\{\alpha_{DAB}\left(724554240\;n^{2}\;m-36822016\;n^{2}+362277120\;n\;m^{2}+730158328\;n\;m-20749916\;n-362277120\;m^{3}+648108164\;m^{2}+58498204\;m-1579204\right)/\left(2046528\;m+2046528\;n+223839\right),\alpha_{DAB}\left(101658624\;n^{2}\;m-5336320\;n^{2}+50829312\;n\;m^{2}+102104896\;n\;m-3094784\;n-50829312\;m^{3}\;+90762848\;m^{2}+8024128\;m-252670\right)/\left(179520\;m+179520\;n+19635\right),\alpha_{DAB}\left(285780480\;n^{2}\;m-18658816\;n^{2}+142890240\;n\;m^{2}+279720088\;n\;m-12919676\;n-142890240\;m^{3}+251493044\;m^{2}+18337564\;m-1359754\right)/\left(682176\;m+682176\;n+74613\right)\right\}$

Result 4 *For the bi-trapezium configuration* BZ *of dimension* (n,m) *, the scaled face reverse-degree indices are given by*

1.
$F^{*}M_{1}^{r_{k}}\left(BZ\right)=\left\{\alpha_{BZ}\left(59765760\;n^{2}\;m-2224000\;n^{2}+29882880\;n\;m^{2}+63535552\;n\;m-1306760\;n-29882880\;m^{3}+53432864\;m^{2}+6911032\;m-126040\right)/\left(193200\;m+193200\;n+26565\right),\;\alpha_{BZ}\left(96860160\;n^{2}\;m-3769600\;n^{2}+48430080\;n\;m^{2}+102639232\;n\;m-2292080\;n-48430080\;m^{3}+86431424\;m^{2}+11026192\;m-232300\right)/\left(193200\;m+193200\;n+26565\right),\alpha_{BZ}\left(65940480\;n^{2}\;m-\;3280000\;n^{2}+32970240\;n\;m^{2}+68447296\;n\;m-2313080\;n-32970240\;m^{3}+58127072\;m^{2}+6753736\;m-279220\right)/\left(193200\;m+193200\;n+26565\right)\right\}$

2.
$F^{*}M_{2}^{r_{k}}\left(BZ\right)=\left\{\alpha_{BZ}\left(45610240\;n^{2}\;m-1409600\;n^{2}+22805120\;n\;m^{2}+49062448\;n\;m-658420\;n-22805120\;m^{3}+41064936\;m^{2}+5612988\;m-45770\right)/\left(193200\;m+193200\;n+26565\right),\alpha_{BZ}\left(24784640\;n^{2}\;m-881280\;n^{2}+12392320\;n\;m^{2}+26429968\;n\;m-491568\;n-12392320\;m^{3}+22199416\;m^{2}+2916320\;m-44988\right)/\left(38640\;m+38640\;n+5313\right),\alpha_{BZ}\left(48463360\;n^{2}\;m-2718400\;n^{2}+24231680\;n\;m^{2}+49690272\;n\;m-1882580\;n-24231680\;m^{3}+42413104\;m^{2}+4781132\;m-250930\right)/\left(193200\;m+193200\;n+26565\right)\right\}$

3.
$F^{*}HM^{r_{k}}\left(BZ\right)=\left\{\alpha_{BZ}\left(196328960\;n^{2}\;m-6400000\;n^{2}+98164480\;n\;m^{2}+210524192\;n\;m-3266480\;n-98164480\;m^{3}+176431344\;m^{2}+23728752\;m-257140\right)/\left(193200\;m+193200\;n+26565\right),\;\alpha_{BZ}\left(101916160\;n^{2}\;m-3677440\;n^{2}+50958080\;n\;m^{2}+108574752\;n\;m-2092832\;n-50958080\;m^{3}+91231984\;m^{2}+11920640\;m-194764\right)/\left(38640\;m+38640\;n+5313\right),\alpha_{BZ}\left(249405440n^{2}\;m-13920000\;n^{2}+124702720\;n\;m^{2}+255858688\;n\;m-10061520\;n-124702720\;m^{3}+218338816\;m^{2}+24231728\;m-1299960\right)/\left(193200\;m+193200\;n+26565\right)\right\}$

4.
$F^{*}F^{r_{k}}\left(BZ\right)=\left\{\alpha_{BZ}\left(35036160\;n^{2}\;m-1193600\;n^{2}+17518080\;n\;m^{2}+37466432\;n\;m-649880\;n-\;17518080\;m^{3}+31433824\;m^{2}+4167592\;m-55200\right)/\left(64400\;m+64400\;n+8855\right),\alpha_{BZ}\left(52346880\;n^{2}\;m-1914880\;n^{2}+26173440\;n\;m^{2}+55714816\;n\;m-1109696\;n-26173440\;m^{3}+46833152\;m^{2}+6088000\;m-104788\right)/\left(38640\;m+38640\;n+5313\right),\alpha_{BZ}\left(152478720\;n^{2}\;m-8483200\;n^{2}+76239360\;n\;m^{2}+\;156478144\;n\;m-6296360\;n-76239360\;m^{3}+133512608\;m^{2}+14669464\;m-798100\right)/\left(193200\;m+193200\;n+26565\right)\right\}$

5.
$F^{*}A^{r_{k}}\left(BZ\right)=\left\{\alpha_{BZ}\left(29882880\;n^{2}\;m-1112000\;n^{2}+14941440\;n\;m^{2}+31767776\;n\;m-653380\;n-\;14941440\;m^{3}+26716432\;m^{2}+3455516\;m-63020\right)/\left(193200\;m+193200\;n+26565\right),\alpha_{BZ}\left(48430080\;n^{2}\;m\;-1884800\;n^{2}+24215040\;n\;m^{2}+51319616\;n\;m-1146040\;n-24215040\;m^{3}+43215712\;m^{2}+5513096\;m-116150\right)/\left(193200\;m+193200\;n+26565\right),\alpha_{BZ}\left(32970240\;n^{2}\;m-1640000\;n^{2}+16485120\;n\;m^{2}+\;34223648\;n\;m-1156540\;n-16485120\;m^{3}+29063536\;m^{2}+3376868\;m-139610\right)/\left(193200\;m+193200\;n+26565\right)\right\}$

6.
$F^{*}BM^{r_{k}}\left(BZ\right)=\left\{\alpha_{BZ}\left(21075200\;n^{2}\;m-726720\;n^{2}+10537600\;n\;m^{2}+22519600\;n\;m-393036\;n-10537600\;m^{3}+18899560\;m^{2}+2504804\;m-34362\right)/\left(38640\;m+38640\;n+5313\right),\alpha_{BZ}\left(31540480\;n^{2}\;m-1168000\;n^{2}+15770240\;n\;m^{2}+33541296\;n\;m-678560\;n-15770240\;m^{3}+28204072\;m^{2}+3658256\;m-65320\right)/\left(27600\;m+27600\;n+3795\right),\alpha_{BZ}\left(4974080\;n^{2}\;m-260800\;n^{2}+2487040\;n\;m^{2}+5136416\;n\;m-182420\;n-2487040\;m^{3}+4371312\;m^{2}+501516\;m-23050\right)/\left(8400\;m+8400\;n+1155\right)\right\}$

7.
$F^{*}TM^{r_{k}}\left(BZ\right)=\left\{\alpha_{BZ}\left(150718720\;n^{2}\;m-4990400\;n^{2}+75359360\;n\;m^{2}+161461744\;n\;m-2608060\;n-75359360\;m^{3}+135366408\;m^{2}+18115764\;m-211370\right)/\left(193200\;m+193200\;n+26565\right),\;\alpha_{BZ}\left(77131520\;n^{2}\;m-2796160\;n^{2}+38565760\;n\;m^{2}+82144784\;n\;m-1601264\;n-38565760\;m^{3}+69032568\;m^{2}+9004320\;m-149776\right)/\left(38640\;m+38640\;n+5313\right),\alpha_{BZ}\left(200942080\;n^{2}\;m-11201600\;n^{2}+100471040\;n\;m^{2}+206168416\;n\;m-8178940\;n-100471040\;m^{3}+175925712\;m^{2}+19450596\;m-1049030\right)/\left(193200\;m+193200\;n+26565\right)\right\}$

8.
$F^{*}BMA^{r_{k}}\left(BZ\right)=\left\{\alpha_{BZ}\left(193987840\;n^{2}\;m-7592000\;n^{2}+96993920\;n\;m^{2}+205477168\;n\;m-4599700\;n\;-96993920\;m^{3}+173059176\;m^{2}+22073628\;m-470810\right)/\left(579600\;m+579600\;n+79695\right),\;\alpha_{BZ}\left(84135680\;n^{2}\;m-3354240\;n^{2}+42067840\;n\;m^{2}+88995856\;n\;m-2068080\;n-42067840\;m^{3}+74997112\;m^{2}+9500576\;m-215004\right)/\left(193200\;m+193200\;n+26565\right),\alpha_{BZ}\left(63120640\;n^{2}\;m-2804800\;n^{2}+31560320\;n\;m^{2}+66190128\;n\;m-1825460\;n-31560320\;m^{3}+55976296\;m^{2}+6853628\;m-208840\right)/\left(193200\;m+193200\;n+26565\right)\right\}$

9.
$F^{*}TMH^{r_{k}}\left(BZ\right)=\left\{\alpha_{BZ}\left(12001280\;n^{2}\;m-340800\;n^{2}+6000640\;n\;m^{2}+12969856\;n\;m-140700\;n-6000640\;m^{3}+10835392\;m^{2}+1509476\;m-6900\right)/\left(9200\;m+9200\;n+1265\right),\alpha_{BZ}\left(1021826560\;n^{2}\;m-34073600\;n^{2}+510913280\;n\;m^{2}+1094180512\;n\;m-17820880\;n-510913280\;m^{3}+917502384\;m^{2}+122680272\;m-1468550\right)/\left(193200\;m+193200\;n+26565\right),\alpha_{BZ}\left(395932160\;n^{2}\;m-23953600\;n^{2}+197966080\;n\;m^{2}+402465632\;n\;m-17244500\;n-197966080\;m^{3}+344758224\;m^{2}+37196172\;m-2347150\right)/\left(193200\;m+193200\;n+26565\right)\right\}$

10.
$F^{*}TMA^{r_{k}}\left(BZ\right)=\left\{\alpha_{BZ}\left(275889920\;n^{2}\;m-10388800\;n^{2}+137944960\;n\;m^{2}+293047184\;n\;m-6196820\;n-137944960\;m^{3}+246533688\;m^{2}+31738044\;m-604210\right)/\left(579600\;m+579600\;n+79695\right),\alpha_{BZ}\left(146679040\;n^{2}\;m-5730560\;n^{2}+73339520\;n\;m^{2}+155386288\;n\;m-3501400\;n-73339520\;m^{3}+130864296\;m^{2}+16666968\;m-355856\right)/\left(193200\;m+193200\;n+26565\right),\alpha_{BZ}\left(105854720\;n^{2}\;m-\;5300800\;n^{2}+52927360\;n\;m^{2}+109808144\;n\;m-3786020\;n-52927360\;m^{3}+93276408\;m^{2}+10769004\;m-455860\right)/\left(193200\;m+193200\;n+26565\right)\right\}$

The scaled face reverse-degree topological indices of DAB and BZ are computed using Results 3 and 4, and the corresponding values are summarized in Tables 6–8.

**TABLE 6:** Scaled face reverse-degree (k=1) topological indices of DAB and BZ for varying (n,m) .

$F^{*}TI^{r_{1}}$	$F^{*}M_{1}^{r_{1}}$	$F^{*}M_{2}^{r_{1}}$	$F^{*}HM^{r_{1}}$	$F^{*}F^{r_{1}}$	$F^{*}A^{r_{1}}$	$F^{*}BM^{r_{1}}$	$F^{*}TM^{r_{1}}$	$F^{*}BMA^{r_{1}}$	$F^{*}TMH^{r_{1}}$	$F^{*}TMA^{r_{1}}$
DAB(1,1)	2402.3311	1835.1577	7901.3088	4230.9934	1201.1656	4237.4889	6066.1511	2595.7193	10150.4431	3696.9430
DAB(2,1)	4045.1763	3078.1173	13267.7634	7111.5288	2022.5881	7123.2936	10189.6461	4375.3724	17000.4682	6226.9801
DAB(2,2)	6530.3675	4953.1271	21369.3569	11463.1026	3265.1838	11483.4947	16416.2297	7069.7091	27322.3142	10055.0260
DAB(3,1)	5688.0968	4321.2039	18634.6982	9992.2903	2844.0484	10009.3007	14313.4943	6155.0680	23851.3688	8757.1256
DAB(3,2)	9857.9842	7460.3464	32206.6453	17285.9525	4928.9921	17318.3306	24746.2989	10678.7821	41117.0548	15181.1862
DAB(4,1)	7331.0457	5564.3379	24001.8130	12873.1371	3665.5229	12895.3836	18437.4750	7934.7793	30702.5961	11287.3121
DAB(4,2)	13185.6744	9967.7017	43044.4265	23109.0231	6592.8372	23153.3761	33076.7248	14287.9006	54912.7218	20307.4482
DAB(4,3)	17355.7827	13107.2563	56617.8606	30403.3480	8677.8913	30463.0390	43510.6043	18811.7521	72181.2118	26731.8132
DAB(5,1)	8974.0085	6807.4949	29369.0153	15754.0255	4487.0043	15781.5034	22561.5204	9714.4983	37553.9820	13817.5187
DAB(5,2)	16513.3951	12475.1129	53882.4111	28932.1854	8256.6976	28988.5080	41407.2982	17897.0376	68708.7700	25433.7526
DAB(5,3)	22368.3275	16879.0455	72927.0736	39168.9826	11184.1638	39247.3731	56048.0281	24250.3485	92922.7645	34454.3065
DAB(5,4)	26538.4931	20018.7062	86500.8916	46463.4793	13269.2465	46557.1993	66482.1854	28774.2354	110191.9767	40878.7508
BZ(1,1)	3239.5585	2506.0827	10746.8410	5734.6756	1619.7793	5745.6412	8240.7583	3491.3609	13919.8339	4979.7562
BZ(2,1)	5440.8483	4196.9082	18012.3612	9618.5448	2720.4241	9637.7565	13815.4530	5868.3027	23286.7217	8365.3938
BZ(2,2)	8763.4301	6743.3403	28960.9707	15474.2902	4381.7150	15506.7703	22217.6305	9458.4468	37381.1866	13476.4134
BZ(3,1)	7642.1901	5887.8225	25278.2154	13502.5704	3821.0951	13530.0126	19390.3929	8245.2742	32654.2203	11751.1061
BZ(3,2)	13207.3672	10145.5035	43593.1087	23302.1017	6603.6836	23352.8708	33447.6052	14261.8345	56204.0721	20312.8999
BZ(4,1)	9843.5519	7578.7703	32544.1960	17386.6555	4921.7759	17422.3221	24965.4258	10622.2568	42021.9493	15136.8469
BZ(4,2)	17651.3598	13547.7693	58225.6180	31130.0795	8825.6799	31199.1291	44677.8487	19065.2564	75027.6553	27149.4632
BZ(4,3)	23216.7053	17805.7635	76541.6428	40930.1158	11608.3526	41022.4688	58735.8793	25081.9212	98579.6389	35711.4894
BZ(5,1)	12044.9233	9269.7343	39810.2385	21270.7699	6022.4617	21314.6576	30540.5042	12999.2448	51389.7906	18522.6019
BZ(5,2)	22095.3755	16950.0772	72858.2809	38958.1264	11047.6877	39045.4527	55908.2037	23868.6924	93851.5266	33986.0586
BZ(5,3)	29903.4186	22919.5145	98541.2847	52702.2557	14951.7093	52822.9331	75621.7702	32311.8382	126860.2149	45998.9990
BZ(5,4)	35468.8082	27177.5904	116857.6056	62502.4247	17734.4041	62646.3987	89680.0152	38328.5302	150412.7550	54561.0863

**TABLE 7:** Scaled face reverse-degree (k=2) topological indices of DAB and BZ for varying (n,m) .

$F^{*}TI^{r_{2}}$	$F^{*}M_{1}^{r_{2}}$	$F^{*}M_{2}^{r_{2}}$	$F^{*}HM^{r_{2}}$	$F^{*}F^{r_{2}}$	$F^{*}A^{r_{2}}$	$F^{*}BM^{r_{2}}$	$F^{*}TM^{r_{2}}$	$F^{*}BMA^{r_{2}}$	$F^{*}TMH^{r_{2}}$	$F^{*}TMA^{r_{2}}$
DAB(1,1)	3890.3311	4981.4889	20486.6333	10523.6556	1945.1656	8871.8200	15505.1445	3377.0978	41111.0475	5891.5645
DAB(2,1)	6557.1763	8379.2936	34472.4685	17713.8814	3278.5881	14936.4698	26093.1749	5695.0587	69063.0526	9931.2938
DAB(2,2)	10594.3675	13515.4947	55618.8270	28587.8376	5297.1838	24109.8622	42103.3323	9205.4989	111275.2331	16047.2361
DAB(3,1)	9224.0968	11777.3007	48459.0853	24904.4839	4612.0484	21001.3975	36681.7846	8013.0601	97017.3458	13971.1334
DAB(3,2)	16001.9842	20390.3306	83926.5819	43145.9208	8000.9921	36392.3147	63536.2514	13908.4661	167750.2504	24239.5022
DAB(4,1)	11891.0457	15175.3836	62445.9958	32095.2285	5945.5229	27066.4293	47270.6122	10331.0767	124972.4963	18011.0147
DAB(4,2)	21409.6744	27265.3761	112235.1239	57704.3718	10704.8372	48675.0504	84969.7479	18611.4752	224227.6209	32431.8735
DAB(4,3)	28187.7827	35879.0390	147704.9912	75946.9133	14093.8913	64066.8216	111825.9522	24507.0078	294967.6289	42700.5575
DAB(5,1)	14558.0085	18573.5034	76433.0493	39286.0426	7279.0043	33131.5119	57859.5460	12649.1007	152928.0637	22050.9164
DAB(5,2)	26817.3951	34140.5080	140543.9916	72262.9756	13408.6976	60957.9031	106403.4836	23314.5016	280705.9629	40624.2886
DAB(5,3)	36336.3275	46231.3731	190336.3837	97873.6376	18168.1638	82567.7006	144105.0107	31595.0746	379970.8769	55045.5804
DAB(5,4)	43114.4931	54845.1993	225806.8640	116116.4654	21557.2465	97959.6924	170961.6647	37490.6399	450712.7184	65314.3463
BZ(1,1)	5231.5585	6741.6412	27689.0751	14205.7926	2615.7793	11973.1997	20947.4338	4535.5282	55846.8670	7919.5888
BZ(2,1)	8792.8483	11313.7565	46479.7542	23852.2413	4396.4241	20106.6047	35165.9978	7625.9513	93632.5336	13311.7452
BZ(2,2)	14171.4301	18210.7703	74830.6910	38409.1503	7085.7150	32382.2004	56619.9207	12294.9541	150587.7084	21455.9061
BZ(3,1)	12354.1901	15886.0126	65270.9759	33498.9506	6177.0951	28240.2028	49384.9633	10716.4025	131419.7919	18703.9777
BZ(3,2)	21367.3672	27432.8708	112742.5776	57876.8361	10683.6836	48800.2380	85309.7069	18542.5742	226714.4929	32352.1603
BZ(4,1)	15915.5519	20458.3221	84062.4034	43145.7592	7957.7759	36373.8739	63604.0813	13806.8644	169207.6524	24096.2393
BZ(4,2)	28563.3598	36655.1291	150655.0572	77344.7991	14281.6799	65218.4889	113999.9282	24790.2258	302843.0501	43248.4938
BZ(4,3)	37576.7053	48202.4688	198128.4639	101723.5264	18788.3526	85779.1740	149925.9951	32616.4938	398143.1561	56896.9168
BZ(5,1)	19476.9233	25030.6576	102853.9317	52792.6165	9738.4617	44507.5809	77823.2741	16897.3315	206995.8074	29488.5151
BZ(5,2)	35759.3755	45877.4527	188567.7828	96812.8774	17879.6877	81636.8282	142690.3301	31037.8905	378972.3413	54144.8604
BZ(5,3)	48407.4186	62074.9331	255162.9590	131013.0928	24203.7093	110482.3516	193088.0259	42021.3866	512615.2958	73297.4505
BZ(5,4)	57420.8082	73622.3987	302636.8385	155392.0412	28710.4041	131043.2069	229014.4399	49847.6797	607916.8156	86945.9367

**TABLE 8:** Scaled face reverse-degree (k=3) topological indices of DAB and BZ for varying (n,m) .

$F^{*}TI^{r_{3}}$	$F^{*}M_{1}^{r_{3}}$	$F^{*}M_{2}^{r_{3}}$	$F^{*}HM^{r_{3}}$	$F^{*}F^{r_{3}}$	$F^{*}A^{r_{3}}$	$F^{*}BM^{r_{3}}$	$F^{*}TM^{r_{3}}$	$F^{*}BMA^{r_{3}}$	$F^{*}TMH^{r_{3}}$	$F^{*}TMA^{r_{3}}$
DAB(1,1)	2635.3378	1917.3198	9911.9906	6077.3510	1317.6689	4552.6575	7994.6708	2525.3299	15634.2402	4233.3456
DAB(2,1)	4469.6475	3260.7065	16864.0027	10342.5898	2234.8237	7730.3539	13603.2963	4269.1766	26657.4177	7182.1183
DAB(2,2)	7259.2650	5308.8333	27462.7264	16845.0598	3629.6325	12568.0982	22153.8931	6915.2083	43494.0937	11667.3216
DAB(3,1)	6303.8065	4603.8480	23814.9218	14607.2258	3151.9032	10907.6544	19211.0738	6012.9620	37678.1065	10130.6509
DAB(3,2)	11004.0317	8061.5442	41707.2152	25584.1268	5502.0159	19065.5759	33645.6710	10463.3861	66142.4646	17688.6773
DAB(4,1)	8137.9086	5946.8946	30765.4236	18871.6344	4068.9543	14084.8032	24818.5290	7756.7237	48697.8370	13079.0935
DAB(4,2)	14748.6513	10814.0632	55950.7315	34322.6051	7374.3256	25562.7145	45136.6683	14011.5158	88788.7851	23709.7868
DAB(4,3)	19448.4347	14271.1987	73840.1361	45297.7388	9724.2174	33719.6334	59568.9375	18461.7997	117247.1109	31267.0697
DAB(5,1)	9971.9830	7289.8944	37715.7206	23135.9319	4985.9915	17261.8773	30425.8263	9500.4736	59717.0953	16027.4924
DAB(5,2)	18493.2098	13566.5003	70193.8397	43060.8390	9246.6049	32059.7101	56627.3394	17559.6251	111434.2368	29730.7944
DAB(5,3)	25103.3450	18432.9070	95375.1937	58509.3798	12551.6725	43536.2519	76942.2868	23814.2267	151516.9630	40360.4632
DAB(5,4)	29803.0138	21889.8939	113263.8431	69484.0553	14901.5069	51692.9077	91373.9492	28264.4735	179973.6994	47917.5542
BZ(1,1)	3480.8830	2528.7455	13005.0228	7947.5318	1740.4415	6009.6285	10476.2773	3371.1864	20461.0544	5582.5795
BZ(2,1)	5878.3035	4279.1503	22015.5144	13457.2139	2939.1517	10157.4537	17736.3642	5678.7876	34695.2642	9429.8194
BZ(2,2)	9513.1399	6939.0603	35706.6801	21828.5595	4756.5699	16452.2002	28767.6198	9170.7651	56360.4722	15263.5147
BZ(3,1)	8275.6198	6029.3888	31025.2567	18966.4790	4137.8099	14305.0086	24995.8678	7986.3472	48927.7798	13276.8923
BZ(3,2)	14385.2656	10508.3420	54077.7462	33061.0623	7192.6328	24893.6076	43569.4043	13847.0855	85455.1654	23083.4456
BZ(4,1)	10672.8963	7779.5625	40034.7103	24475.5852	5336.4482	18452.4588	32255.1478	10293.8906	63159.6373	17123.9020
BZ(4,2)	19257.2804	14077.4789	72448.0792	44293.1215	9628.6402	33334.7593	58370.6004	18523.3697	114548.3124	30903.1910
BZ(4,3)	25366.5895	18556.0013	95498.3603	58386.3578	12683.2947	43922.5907	76942.3591	24384.0003	151071.0731	40709.1786
BZ(5,1)	13070.1534	9529.7040	49044.0216	29984.6135	6535.0767	22599.8574	39514.3175	12601.4260	77391.1689	20970.8808
BZ(5,2)	24129.2490	17646.5542	90818.1045	55524.9962	12064.6245	41775.8032	73171.5503	23199.6385	143640.8048	38722.8596
BZ(5,3)	32713.1629	23943.8724	123228.3963	75340.6515	16356.5814	56657.0353	99284.5239	31428.9681	195023.0491	52501.3576
BZ(5,4)	38822.3835	28422.2794	146278.0929	89433.5341	19411.1918	67244.6629	117855.8135	37289.5699	231544.5775	62307.1972

### 3.3 Existence of isentropic structures

The term isentropic, meaning constant entropy, has been of long-standing interest in thermodynamics and has led to diverse concepts such as isentropic analysis, isentropic efficiency, and isentropic flow. In the context of topological analysis, isentropic structures are defined as systems that attain equal entropy values even in the presence of variations in topology, geometry, or composition. The recognition of such structures is significant, as it reveals underlying structural similarity, indicates equivalent information content, and enables the classification of networks into entropy-equivalent families. These measures provide an effective framework for quantifying structural complexity and information distribution in molecular and networked systems (Anand and Bianconi, 2009; Bonchev, 1983; Mowshowitz, 1968). They have been successfully applied to both organic and inorganic materials, where entropy-based approaches contribute to the analysis of framework organization and support rational material design (Jacob et al., 2023; Rahul et al., 2022). These entropy formulations are constructed through additive and multiplicative self-powered transformations of the underlying indices, thereby incorporating both frequency and magnitude information associated with bond partitions. Recent advancements have incorporated reverse-degree modifications into entropy measures. As a result reverse-degree-based entropy of TP-COF takes the form

$$I_{TI^{r_{k}}}\left(\text{TP}-\text{COF}\right)=\log\left(TI^{r_{k}}\left(\text{TP}-\text{COF}\right)\right)-\frac{1}{TI^{r_{k}}\left(\text{TP}-\text{COF}\right)}\log\left(TI^{p*}\left(\text{TP}-\text{COF}\right)\right).$$

where, $TI^{p*}\left(TP-COF\right)$ is the multiplicative self-powered transformation defined as $TI^{p*}\left(TP-COF\right)=\prod_{\left(x,y\right)\in D\left(TP-COF\right)}d\left(x,y\right)\;TI^{r_{k}}{\left(x,y\right)}^{TI^{r_{k}}\left(x,y\right)}$ . Explicit mathematical expressions for the entropy measures can be derived from the corresponding topological and self-powered indices. However, the resulting formulations are complex and difficult to present concisely. Therefore, we focus on the numerical evaluation of the entropy values for DAB and BZ . The computed values are presented in Tables 9–11.

**TABLE 9:** Reverse-degree (k=1) based entropy measures of DAB and BZ for varying (n,m) .

$I_{TI^{r_{1}}}$	$I_{M_{1}^{r_{1}}}$	$I_{M_{2}^{r_{1}}}$	$I_{HM^{r_{1}}}$	$I_{F^{r_{1}}}$	$I_{A^{r_{1}}}$	$I_{BM^{r_{1}}}$	$I_{TM^{r_{1}}}$	$I_{BMA^{r_{1}}}$	$I_{TMH^{r_{1}}}$	$I_{TMA^{r_{1}}}$
DAB(3,2)	9.1912	8.9208	10.3787	9.7519	8.4968	9.7588	10.1137	9.2746	10.6281	9.6216
DAB(4,3)	9.7578	9.4856	10.9437	10.3172	9.0638	10.3244	10.6788	9.8420	11.1918	10.1882
DAB(4,4)	9.8919	9.6195	11.0777	10.4512	9.1979	10.4584	10.8127	9.9761	11.3257	10.3223
DAB(6,2)	9.8919	9.6195	11.0777	10.4512	9.1979	10.4584	10.8127	9.9761	11.3257	10.3223
DAB(5,1)	9.0973	8.8291	10.2866	9.6593	8.4027	9.6658	10.0214	9.1798	10.5375	9.5276
DAB(5,4)	10.1829	9.9096	11.3679	10.7416	9.4892	10.7490	11.1030	10.2676	11.6153	10.6133
DAB(7,7)	10.8645	10.5900	12.0485	11.4224	10.1710	11.4299	11.7836	10.9496	12.2950	11.2949
DAB(11,3)	10.8645	10.5900	12.0485	11.4224	10.1710	11.4299	11.7836	10.9496	12.2950	11.2949
DAB(10,10)	11.5122	11.2367	12.6953	12.0694	10.8188	12.0771	12.4305	11.5977	12.9412	11.9426
DAB(16,4)	11.5122	11.2367	12.6953	12.0694	10.8188	12.0771	12.4305	11.5977	12.9412	11.9426
DAB(13,13)	11.9996	11.7236	13.1822	12.5564	11.3063	12.5643	12.9175	12.0853	13.4277	12.4300
DAB(21,5)	11.9996	11.7236	13.1822	12.5564	11.3063	12.5643	12.9175	12.0853	13.4277	12.4300
BZ(3,2)	9.4710	9.2036	8.7767	10.6581	10.0283	10.0398	10.3920	9.5587	10.9090	9.9002
BZ(4,3)	10.0359	9.7672	11.2218	10.5922	9.3421	10.6040	10.9558	10.1242	11.4718	10.4651
BZ(4,4)	10.1698	9.9010	9.4760	11.3556	10.7261	10.7378	11.0896	10.2581	11.6056	10.5990
BZ(6,2)	10.1698	9.9010	9.4760	11.3556	10.7261	10.7378	11.0896	10.2581	11.6056	10.5990
BZ(5,1)	9.3787	9.1131	8.6844	10.5672	9.9370	9.9483	10.3010	9.4657	10.8193	9.8079
BZ(5,4)	10.4601	10.1905	11.6452	11.0159	9.7664	11.0277	11.3793	10.5487	11.8947	10.8893
BZ(7,7)	11.1407	10.8702	10.4472	12.3250	11.6958	11.7078	12.0591	11.2296	12.5739	11.5699
BZ(11,3)	11.1407	10.8702	10.4472	12.3250	11.6958	11.7078	12.0591	11.2296	12.5739	11.5699
BZ(10,10)	11.7875	11.5164	11.0942	12.9713	12.3422	12.3543	12.7054	11.8768	13.2196	12.2168
BZ(16,4)	11.7875	11.5164	11.0942	12.9713	12.3422	12.3543	12.7054	11.8768	13.2196	12.2168
BZ(13,13)	12.2745	12.0029	11.5812	13.4579	12.8289	12.8411	13.1920	12.3639	13.7059	12.7038
BZ(21,5)	12.2745	12.0029	11.5812	13.4579	12.8289	12.8411	13.1920	12.3639	13.7059	12.7038

**TABLE 10:** Reverse-degree (k=2) based entropy measures of DAB and BZ for varying (n,m) .

$I_{TI^{r_{2}}}$	$I_{M_{1}^{r_{2}}}$	$I_{M_{2}^{r_{2}}}$	$I_{HM^{r_{2}}}$	$I_{F^{r_{2}}}$	$I_{A^{r_{2}}}$	$I_{BM^{r_{2}}}$	$I_{TM^{r_{2}}}$	$I_{BMA^{r_{2}}}$	$I_{TMH^{r_{2}}}$	$I_{TMA^{r_{2}}}$
DAB(3,2)	9.6766	9.9218	11.3342	10.6674	8.9829	10.4997	11.0554	9.5379	12.0281	10.0913
DAB(4,3)	10.2438	10.4878	11.9006	11.2338	9.5503	11.0663	11.6218	10.1055	12.5938	10.6585
DAB(4,4)	10.3778	10.6218	12.0347	11.3678	9.6843	11.2003	11.7558	10.2395	12.7279	10.7925
DAB(6,2)	10.3778	10.6218	12.0347	11.3678	9.6843	11.2003	11.7558	10.2395	12.7279	10.7925
DAB(5,1)	9.5820	9.8284	11.2406	10.5738	8.8883	10.4058	10.9618	9.4428	11.9355	9.9966
DAB(5,4)	10.6692	10.9126	12.3256	11.6588	9.9758	11.4914	12.0468	10.5311	13.0184	11.0839
DAB(7,7)	11.3511	11.5938	13.0070	12.3401	10.6578	12.1729	12.7281	11.2133	13.6993	11.7658
DAB(11,3)	11.3511	11.5938	13.0070	12.3401	10.6578	12.1729	12.7281	11.2133	13.6993	11.7658
DAB(10,10)	11.9990	12.2412	13.6545	12.9877	11.3058	12.8206	13.3756	11.8614	14.3463	12.4138
DAB(16,4)	11.9990	12.2412	13.6545	12.9877	11.3058	12.8206	13.3756	11.8614	14.3463	12.4138
DAB(13,13)	12.4866	12.7284	14.1417	13.4750	11.7934	13.3079	13.8629	12.3491	14.8334	12.9014
DAB(21,5)	12.4866	12.7284	14.1417	13.4750	11.7934	13.3079	13.8629	12.3491	14.8334	12.9014
BZ(3,2)	9.9581	10.2033	9.2645	11.6146	10.9466	10.7813	11.3354	9.8215	12.3083	10.3724
BZ(4,3)	10.5235	10.7678	12.1794	11.5113	9.8300	11.3462	11.9001	10.3871	12.8726	10.9377
BZ(4,4)	10.6574	10.9016	9.9640	12.3133	11.6452	11.4801	12.0340	10.5210	13.0065	11.0716
BZ(6,2)	10.6574	10.9016	9.9640	12.3133	11.6452	11.4801	12.0340	10.5210	13.0065	11.0716
BZ(5,1)	9.8654	10.1115	9.1717	11.5226	10.8545	10.6890	11.2434	9.7284	12.2171	10.2796
BZ(5,4)	10.9479	11.1917	12.6034	11.9353	10.2545	11.7703	12.3242	10.8117	13.2963	11.3621
BZ(7,7)	11.6287	11.8720	10.9354	13.2838	12.6157	12.4509	13.0046	11.4927	13.9764	12.0430
BZ(11,3)	11.6287	11.8720	10.9354	13.2838	12.6157	12.4509	13.0046	11.4927	13.9764	12.0430
BZ(10,10)	12.2758	12.5186	11.5825	13.9306	13.2625	13.0977	13.6513	12.1399	14.6228	12.6901
BZ(16,4)	12.2758	12.5186	11.5825	13.9306	13.2625	13.0977	13.6513	12.1399	14.6228	12.6901
BZ(13,13)	12.7628	13.0054	12.0696	14.4174	13.7494	13.5846	14.1382	12.6271	15.1094	13.1771
BZ(21,5)	12.7628	13.0054	12.0696	14.4174	13.7494	13.5846	14.1382	12.6271	15.1094	13.1771

**TABLE 11:** Reverse-degree (k=3) based entropy measures of DAB and BZ for varying (n,m) .

$I_{TI^{r_{3}}}$	$I_{M_{1}^{r_{3}}}$	$I_{M_{2}^{r_{3}}}$	$I_{HM^{r_{3}}}$	$I_{F^{r_{3}}}$	$I_{A^{r_{3}}}$	$I_{BM^{r_{3}}}$	$I_{TM^{r_{3}}}$	$I_{BMA^{r_{3}}}$	$I_{TMH^{r_{3}}}$	$I_{TMA^{r_{3}}}$
DAB(3,2)	9.3060	9.0367	10.6555	10.1516	8.6118	9.8736	10.4350	9.2615	11.1404	9.7762
DAB(4,3)	9.8772	9.6118	11.2299	10.7252	9.1834	10.4466	11.0091	9.8311	11.7178	10.3474
DAB(4,4)	10.0115	9.7464	11.3644	10.8596	9.3178	10.5810	11.1436	9.9653	11.8525	10.4817
DAB(6,2)	10.0115	9.7464	11.3644	10.8596	9.3178	10.5810	11.1436	9.9653	11.8525	10.4817
DAB(5,1)	9.2067	8.9336	10.5532	10.0499	8.5125	9.7727	10.3329	9.1643	11.0353	9.6770
DAB(5,4)	10.3050	10.0416	11.6593	11.1542	9.6113	10.8752	11.4383	10.2579	12.1488	10.7751
DAB(7,7)	10.9893	10.7282	12.3454	11.8398	10.2959	11.5605	12.1243	10.9412	12.8365	11.4594
DAB(11,3)	10.9893	10.7282	12.3454	11.8398	10.2959	11.5605	12.1243	10.9412	12.8365	11.4594
DAB(10,10)	11.6394	11.3800	12.9968	12.4909	10.9460	12.2112	12.7755	11.5904	13.4891	12.1094
DAB(16,4)	11.6394	11.3800	12.9968	12.4909	10.9460	12.2112	12.7755	11.5904	13.4891	12.1094
DAB(13,13)	12.1281	11.8697	13.4863	12.9803	11.4348	12.7004	13.2650	12.0786	13.9793	12.5981
DAB(21,5)	12.1281	11.8697	13.4863	12.9803	11.4348	12.7004	13.2650	12.0786	13.9793	12.5981
BZ(3,2)	9.5987	9.3688	8.9048	10.9678	10.4504	10.1836	10.7423	9.5556	11.4763	10.0653
BZ(4,3)	10.1671	9.9396	11.5383	11.0204	9.4734	10.7531	11.3125	10.1227	12.0486	10.6336
BZ(4,4)	10.3012	10.0738	9.6076	11.6725	11.1546	10.8872	11.4467	10.2567	12.1830	10.7677
BZ(6,2)	10.3012	10.0738	9.6076	11.6725	11.1546	10.8872	11.4467	10.2567	12.1830	10.7677
BZ(5,1)	9.5026	9.2702	8.8086	10.8696	10.3525	10.0864	10.6442	9.4610	11.3764	9.9691
BZ(5,4)	10.5932	10.3670	11.9655	11.4474	9.8997	11.1797	11.7397	10.5481	12.4769	11.0597
BZ(7,7)	11.2759	11.0511	10.5825	12.6493	12.1310	11.8630	12.4234	11.2300	13.1617	11.7423
BZ(11,3)	11.2759	11.0511	10.5825	12.6493	12.1310	11.8630	12.4234	11.2300	13.1617	11.7423
BZ(10,10)	11.9245	11.7008	11.2312	13.2989	12.7804	12.5121	13.0729	11.8779	13.8121	12.3909
BZ(16,4)	11.9245	11.7008	11.2312	13.2989	12.7804	12.5121	13.0729	11.8779	13.8121	12.3909
BZ(13,13)	12.4124	12.1894	11.7192	13.7873	13.2688	13.0003	13.5613	12.3655	14.3010	12.8789
BZ(21,5)	12.4124	12.1894	11.7192	13.7873	13.2688	13.0003	13.5613	12.3655	14.3010	12.8789

From Tables 9–11, we clearly observe that the configurations pair {(4,4),(6,2)} , {(7,7),(11,3)} , {(10,10),(16,4)} , {(13,13),(21,5)} , and so on, yield similar entropy values, leading to the existence of isentropic structures. Furthermore, since the isentropic dimensional patterns are consistent for both DAB and BZ frameworks, this behavior can be expressed in the general forms {TP-COF(3n−2,3n−2),TP-COF(5n−4,n)},n≥2 . Selected examples illustrating the isentropic correspondence between DAB and BZ are listed in Table 12. It should be noted that the term “isentropic” used in this study is based on graph-theoretic entropy and does not refer to thermodynamic entropy in the classical materials science sense. The entropy considered here is a structural descriptor derived from topological indices, quantifying the distribution of connectivity within the network. Accordingly, two TP-COF structures are termed isentropic if they exhibit equal values of this graph-based entropy, even if their topology differs. Thus, the term indicates structural equivalence in terms of information content rather than thermodynamic behavior.

**TABLE 12:** Bond partitions of isentropic TP-COF(n,m) structures.

Isentropic structures	TP-COF(4,4),TP-COF(6,2)		TP-COF(7,7),TP-COF(11,3)	
Bond classes	DAB	BZ	DAB	BZ
d(2,2)	1476	2052	3840	5352
d(2,3)	4464	5616	11856	14880
d(3,3)	252	540	696	1452

## 4 Spectral descriptors and QSPR analysis

In this section, the spectral characteristics of the considered TP-COF structures are investigated to gain insight into their structural properties and connectivity patterns. However, direct computation through density functional theory (DFT) is computationally demanding, time-consuming, and often challenging for large-scale frameworks. Therefore, we adopt a graph-theoretical approach based on the eigenvalues of the adjacency matrix. In particular, graph energy $\left(E_{\text{TP}-\text{COF}}\right)$ , HOMO-LUMO gap $\left(\Delta_{HL}\right)$ , and spectral diameter (SD) are computed using Python programming through adjacency matrices. The graph energy of a TP-COF comprising r vertices is determined from its spectrum obtained via the adjacency matrix representation. Let $\left\{\lambda_{1},\lambda_{2},\ldots ,\lambda_{r}\right\}$ denote the eigenvalues of this matrix. The corresponding graph energy, is defined as

$$E_{\text{TP}-\text{COF}}=\overset{r}{\underset{i=1}{\sum }}|\lambda_{i}|$$

Furthermore, the HOMO-LUMO gap and spectral diameter are computed from the ordered eigenvalue set. Specifically, the HOMO-LUMO gap is evaluated as the difference between the smallest positive eigenvalue and the largest negative eigenvalue, whereas the spectral diameter is defined as the difference between the maximum and minimum eigenvalues of the spectrum. The computed spectral descriptors presented in Table 13 demonstrate distinct behavioural patterns across the investigated TP-COF structures. In particular, the HOMO–LUMO gap remains constant for each framework, attaining fixed values of $\Delta_{HL}=0.7295$ for DAB and $\Delta_{HL}=0.7492$ for BZ irrespective of the dimensional parameters (n,m) . This invariance indicates structural control of the spectral gap within each class. A similar near-constant trend is observed for the spectral diameter, which exhibits only marginal variation with increasing structural size. In contrast, graph energy displays a clear monotonic growth as the parameters (n,m) increase, reflecting its sensitivity to structural expansion and complexity. Owing to this discriminative behaviour, graph energy is selected as the principal descriptor for subsequent QSPR modelling.

**TABLE 13:** Computed spectral descriptors of TP-COF for varying (n,m) .

TP-COF	DAB			BZ		
(n,m)	$E_{DAB}$	SD	$\Delta_{HL}$	$E_{BZ}$	SD	$\Delta_{HL}$
(1,1)	888.8125	4.8424	0.7295	1190.7833	4.8423	0.7492
(2,1)	1498.8752	4.8429	0.7295	2002.1599	4.8426	0.7492
(2,2)	2422.7297	4.8433	0.7295	3227.9852	4.8428	0.7492
(3,1)	2108.9379	4.8431	0.7295	2813.5364	4.8427	0.7492
(3,2)	3660.3759	4.8435	0.7295	4868.2593	4.8429	0.7492
(4,1)	2719.0005	4.8431	0.7295	3624.9130	4.8427	0.7492
(4,2)	4898.0222	4.8435	0.7295	6508.5335	4.8430	0.7492
(4,3)	6449.4603	4.8436	0.7295	8563.2564	4.8430	0.7492
(5,1)	3329.0632	4.8431	0.7295	4436.2895	4.8427	0.7492
(5,2)	6135.6685	4.8436	0.7295	8148.8076	4.8430	0.7492
(5,3)	8314.6902	4.8436	0.7295	11032.4281	4.8430	0.7492
(5,4)	9866.1283	4.8437	0.7295	13087.1510	4.8430	0.7492

**FIGURE 7:**
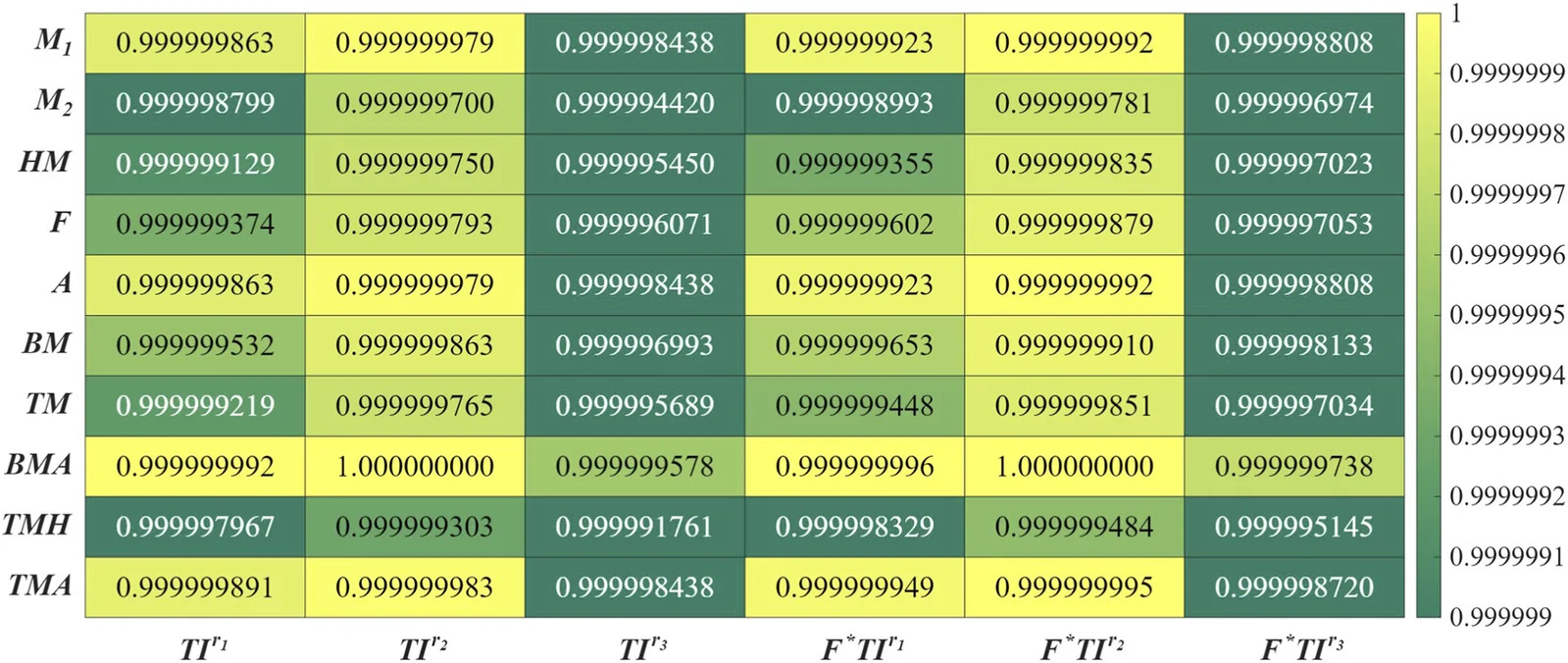
Correlation heatmap of reverse-degree (k=1,2,3) and scaled face reverse-degree (k=1,2,3) indices with graph energy for DAB .

**FIGURE 8:**
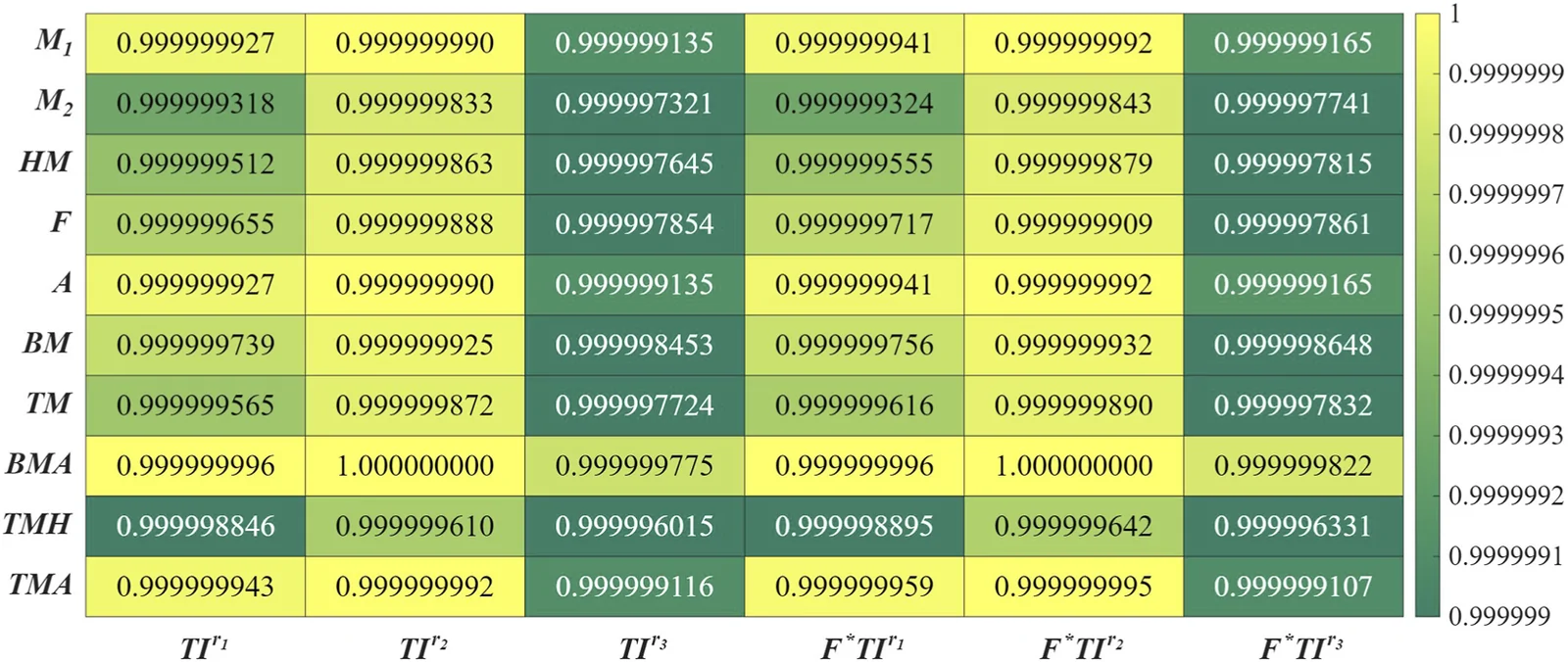
Correlation heatmap of reverse-degree (k=1,2,3) and scaled face reverse-degree (k=1,2,3) indices with graph energy for BZ .

Both univariate and multivariate regression analyses are employed for effective model construction. The univariate regression model is expressed as y=βx+c, where β denotes the slope and c represents the intercept. The multivariate regression model with two independent variables is given by $y=\beta_{1}x_{1}+\beta_{2}x_{2}+c,$ where $\beta_{1}$ and $\beta_{2}$ are the regression coefficients associated with the indices $x_{1}$ and $x_{2}$ , respectively, and c is the intercept. The selection of indices for both univariate and multivariate modelling is initially guided by correlation analysis. For the univariate case, the correlation results are illustrated through heatmaps shown in Figures 7, 8. Based on the best-performing index, univariate regression models are constructed. Similarly, for the multivariate case, all possible pairs of indices are correlated with graph energy using Algorithm 1 for multivariate regression analysis. The most significant index pairs are subsequently employed to develop multivariate regression models. The results of both univariate and multivariate regression analyses are presented in Table 14, where the coefficient of determination ( ${\text{R}}^{2}$ ) and root mean square error (RMSE) are reported to nine and six decimal places, respectively.

**TABLE 14:** Results of univariate and multivariate regression modelling for DAB and BZ .

TI		Equation	*R* ^2^	RMSE
Univariate	$TI^{r_{k}}$	$E_{DAB}=-0.181484+0.263062\left(BMA^{r_{2}}\right)$	1	0.029072
		$E_{BZ}=-0.051313+0.263642\left(BMA^{r_{2}}\right)$	1	0.00822
	$F^{*}TI^{r_{k}}$	$E_{DAB}=0.233282+0.263158\left(F^{*}BMA^{r_{2}}\right)$	0.999999999	0.062665
		$E_{BZ}=0.028083+0.262543\left(F^{*}BMA^{r_{2}}\right)$	1	0.010652
Multivariate	$TI^{r_{k}}$	$E_{DAB}=0.000002+0.563368\left(M_{1}^{r_{1}}\right)-0.250687\left(M_{2}^{r_{1}}\right)$	1	0.000006
		$E_{BZ}=-0.000010+0.758158\left(M_{1}^{r_{3}}\right)-0.539146\left(M_{2}^{r_{3}}\right)$	1	0.000006
	$F^{*}TI^{r_{k}}$	$E_{DAB}=-1.090614-0.330027\left(F^{*}HM^{r_{3}}\right)+0.914001\left(F^{*}BM^{r_{3}}\right)$	1	0.013664
		$E_{BZ}=-0.753171-0.288173\left(F^{*}M_{1}^{r_{3}}\right)+0.650999\left(F^{*}BMA^{r_{3}}\right)$	1	0.010339

Algorithm 1 **Selection of optimal reverse and face reverse degree index pairs for predicting graph energy.**

**Input:** Dataset D containing reverse degree indices $TI^{r_{k}}$ , face reverse degree indices $F^{*}TI^{r_{k}}$ , and
   graph energy $E_{TP-COF}$ ;
**Output:** Best reverse index pair, regression model, and error measures for $E_{\text{TP}-\text{COF}}$ ;
**Preprocessing:**
Load dataset D and remove non-numerical identifiers;
Separate reverse degree indices $TI^{r_{k}}\left(\text{TP}-\text{COF}\right)$ ;
Separate scaled face-reverse degree indices $F^{*}TI^{r_{k}}\left(\text{TP}-\text{COF}\right)$ ;
**Model construction (reverse-degree indices):**
Initialize $R_{\mathrm{best}}^{2}\leftarrow -\infty$ ;
**foreach** *pair* $\left(TI_{x}^{r_{k}},TI_{y}^{r_{k}}\right)$ **do**
  Fit regression model $E_{\text{TP}-\text{COF}}=\beta_{1}TI_{x}^{r_{k}}+\beta_{2}TI_{y}^{r_{k}}+c$ ;
  Compute $R^{2}$ ;
  **if** $R^{2}>R_{\mathrm{best}}^{2}$ **then**
    Update reverse-degree based pair and model
**Model construction (face-reverse degree indices)**
Initialize $R_{\mathrm{best,f}}^{2}\leftarrow -\infty$ ;
**foreach** *pair* $\left(F^{*}TI_{x}^{r_{k}},F^{*}TI_{y}^{r_{k}}\right)$ **do**
  Fit regression model $E_{\text{TP}-\text{COF}}=\beta_{1}F^{*}TI_{x}^{r_{k}}+\beta_{2}F^{*}TI_{y}^{r_{k}}+c$ ;
  Compute $R^{2}$ ;
  **if** $R^{2}>R_{\mathrm{best,f}}^{2}$ **then**
    Update best scaled face-reverse degree based pair and model;
**Error evaluation:**
Compute $\text{RMSE}=\sqrt{\frac{1}{N}\sum e^{2}\;}$ ;
**Output:**
Report best reverse degree index pair;
Report best face reverse degree index pair;
Report regression equations;
Report $R^{2}$ and RMSE;

Table 14 clearly shows that for both DAB and BZ , the association between graph energy and the topological indices follows an almost identical pattern in its correlation behaviour with the indices. In the univariate analysis, the same index consistently exhibits the strongest correlation with energy for both structures. A slight variation is observed only in the case of face reverse-degree-based indices when moving to the multivariate setting. This overall consistency highlights the strong structural similarity between DAB and BZ with respect to their spectral characteristics. In the univariate models, the BMA index dominates the predictive performance. However, in the multivariate framework, the models based on $M_{1}$ and $M_{2}$ indices emerge as the most effective. The multivariate regression models exhibit comparatively low RMSE values, indicating improved predictive accuracy. Among these, the reverse degree-based indices perform particularly well. Specifically, for DAB , the reverse degree descriptor with modification parameter k=1 provides the best performance, whereas for BZ , the descriptor with k=3 performs better. The resulting regression equations corresponding to the best-performing models, along with the standard error ( S.E .) and F -value, are given below.

$$\begin{array}{ll} & E_{DAB}=0.000002+0.563368\left(M_{1}^{r_{1}}\right)-0.250687\left(M_{2}^{r_{1}}\right),\;S.E.=7.12\times 10^{-6},\;F=8.59\times 10^{17},\\ & E_{BZ}=-0.00001+0.758158\left(M_{1}^{r_{3}}\right)-0.539146\left(M_{2}^{r_{3}}\right),\;S.E.=6.48\times 10^{-6},\;F=1.82\times 10^{18}. \end{array}$$

Substituting the index values into these reverse-degree regression equations yields

$$\begin{array}{ll} E_{DAB} & =\frac{2747477953076967655m-78907412229755675n-1413192682653361665m^{2}-78907402762760987}{4503599627370496}\\ & \;+\frac{1413192682653361665mn}{2251799813685248}\\ E_{BZ} & =\frac{913528791220655491m+933255697525240551mn}{1125899906842624}-\frac{4931726576146265n}{281474976710656}\\ & \;-\frac{933255697525240551m^{2}}{2251799813685248}-\frac{10342585920000511442943}{590295810358705651712} \end{array}$$

The low S.E and high F -value indicate the good fit of the model. We examine the two best-performing multivariate models, statistical significance and stability using the p-values, leave-one-out cross-validation coefficient $\left(Q^{2}\right)$ , and the variance inflation factor (VIF). Both models yielded p≤0.01 , confirming statistical significance at the 1 % level. The cross-validation results produced $Q^{2}\approx 1$ , indicating perfect internal predictive agreement within the dataset. However, the VIF values are very high (≥10) , suggesting the presence of multicollinearity between the $M_{1}$ and $M_{2}$ predictors. The elevated VIF values indicate a strong linear association between the $M_{1}$ and $M_{2}$ descriptors, suggesting potential redundancy. This behaviour is not unexpected, as both descriptors are degree-based and derived from closely related degree distributions. Consequently, partial linear dependence may arise, leading to possible coefficient instability in the multivariate regression model. Therefore, while the combined model provides superior fitting performance, caution is required when interpreting the individual regression coefficients, as multicollinearity may inflate statistical measures.

**FIGURE 9:**
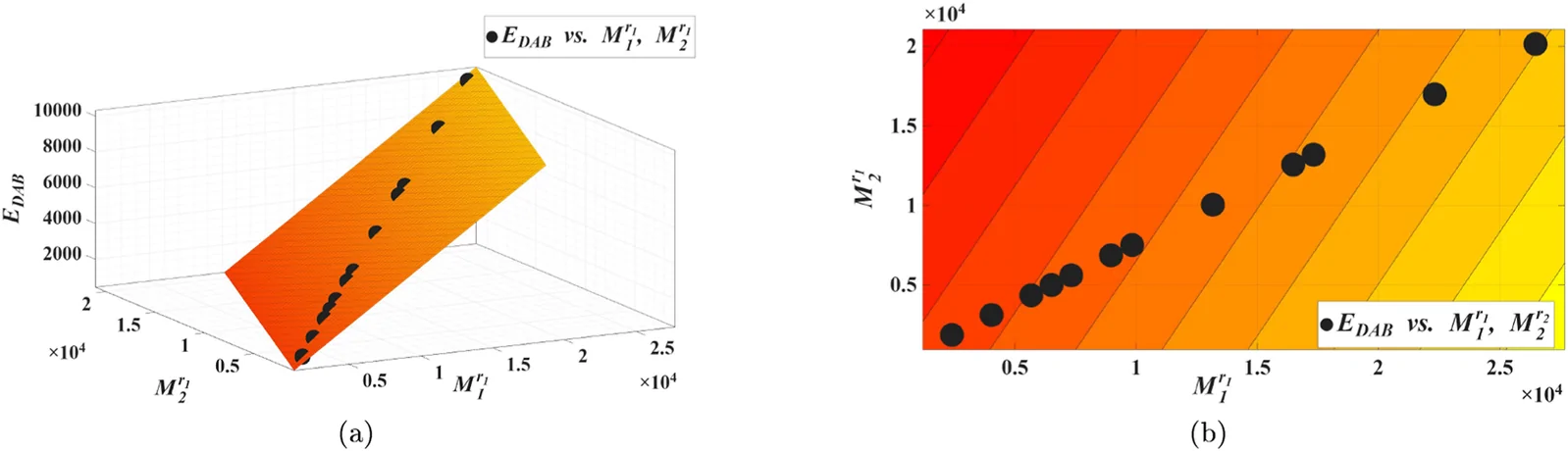
Polynomial surface fitting for energy of DAB : **(a)** surface plot and **(b)** contour plot.

**FIGURE 10:**
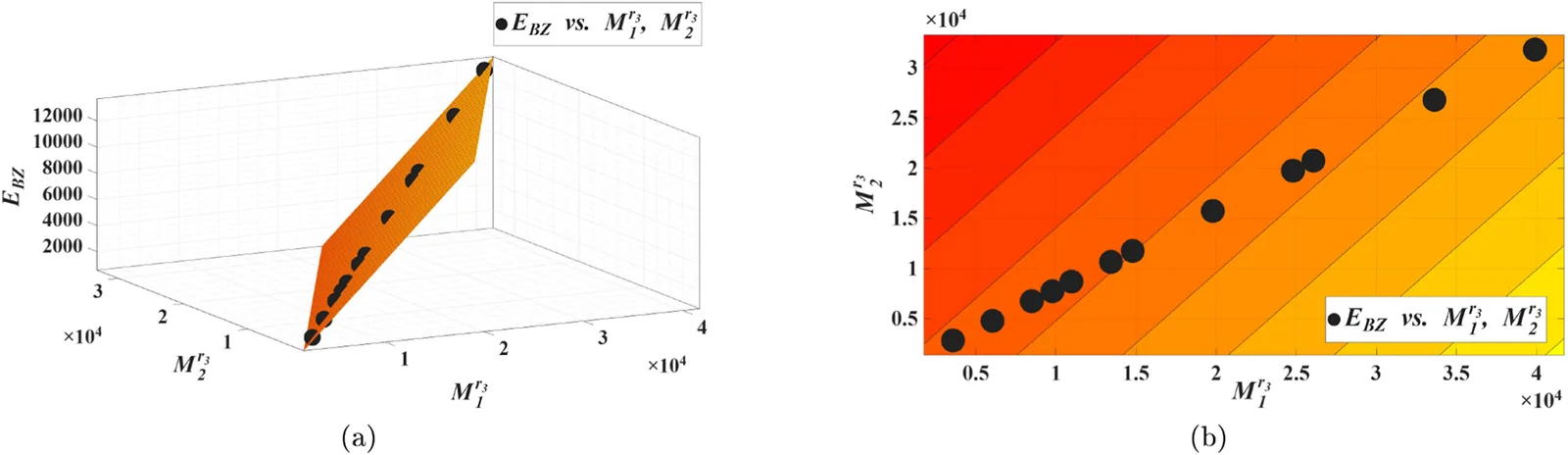
Polynomial surface fitting for energy of BZ : **(a)** surface plot and **(b)** contour plot.

To confirm that the observed perfect predictive ability $\left(Q^{2}\approx 1\right)$ is not merely a consequence of linear dependence between $M_{1}$ and $M_{2}$ , external validation was performed. The regression model was applied to higher-dimensional structures that were not involved in the model fitting process, and the results are presented in Table 15. The sustained predictive accuracy observed in this independent dataset confirms that the excellent performance is not an artefact of multicollinearity but instead reflects a stable and structurally consistent relationship between the selected indices and the energy. To demonstrate the performance of the multivariate models, first-degree polynomial surface fitting were carried out, and the resulting visualizations are depicted in Figures 9, 10.

**TABLE 15:** Actual vs. predicted energy values of DAB and BZ .

TP-COF	$E_{DAB}$		$E_{BZ}$	
(n,m)	Actual	Predicted	Actual	Predicted
(5,5)	10789.98277	10789.98277	14312.97634	14312.97634
(6,3)	10179.92011	10179.92011	13501.59978	13501.59978
(7,4)	14851.75535	14851.75534	19683.28954	19683.28954
(8,2)	9848.60734	9848.60733	13069.62998	13069.62998
(9,5)	23271.57134	23271.57134	30820.84372	30820.8437

Figures 9, 10 clearly illustrate the effectiveness of the polynomial surface fitting for the considered dataset. The fitted surface closely aligns with the observed data points, indicating a strong agreement between the predicted and computed values. The smooth planar nature of the surface suggests a stable and linear relationship between the spectral descriptor and the selected topological descriptors. This confirms the suitability of the proposed multivariate model for predicting graph energy.

### 4.1 Y-scrambling

**FIGURE 11:**
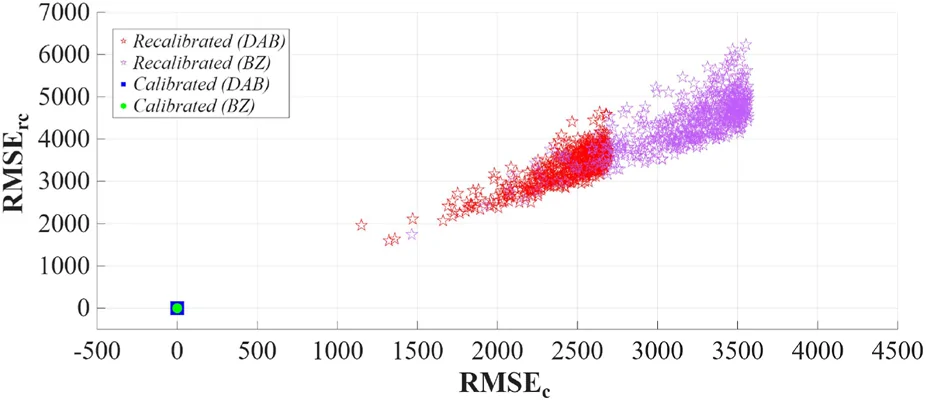
Results of Y-scrambling test for DAB and BZ .

To further validate the robustness and predictive reliability of the proposed QSPR models, a Y-scrambling test is performed. In this procedure, the dependent variable values are randomly shuffled while keeping the independent variables unchanged, and new regression models are constructed for each permutation. The RMSE obtained from each scrambled model is then compared with that of the original model. In this study, the dependent variable is randomly permuted 500 times. For each permutation, the model is recalibrated and the corresponding RMSE is computed. The distribution of RMSE values obtained from the scrambled models is subsequently compared with the RMSE of the original model to assess the presence of chance correlations. The comparative results of the calibrated RMSE ( ${\text{RMSE}}_{\text{c}}$ ) and the recalibrated RMSE ( ${\text{RMSE}}_{\text{rc}}$ ) are graphically illustrated in Figure 11.

From Figure 11, a substantial difference between ${\text{RMSE}}_{\text{c}}$ and ${\text{RMSE}}_{\text{rc}}$ is observed. The significantly lower ${\text{RMSE}}_{\text{c}}$ compared to the ${\text{RMSE}}_{\text{rc}}$ values confirms that the developed QSPR models are not influenced by random correlation and demonstrates their statistical robustness.

## 5 Conclusion

In this work, we carried out a comprehensive structural and spectral investigation of triple-pore covalent organic frameworks using reverse-degree, and scaled face reverse-degree indices. The study provides a systematic mathematical characterization of these frameworks and establishes clear relationships between their structure and spectral behavior. The entropy analysis revealed that the considered TP-COF structures exhibit isentropic behavior, indicating structural regularity and uniform information distribution. From the spectral perspective, graph energy increases monotonically with structural growth, while the HOMO–LUMO gap remains invariant and the spectral diameter shows only marginal variation. The invariance of HOMO–LUMO indicates structural control of the gap within the series in the graph-theoretical model, whereas the monotonic growth of graph energy highlights its suitability as a descriptor for QSPR modelling. Regression analysis further strengthened these observations. Both univariate and multivariate models were constructed to examine the predictive capability of the proposed indices. The multivariate models, particularly those involving the first and second Zagreb indices, produced extremely low RMSE (0.000006) with a leave-one-out cross-validation coefficient close to one. The statistical significance was confirmed through very low p-values, and external validation demonstrated high predictive accuracy. Furthermore, the regression models were validated using a Y-scrambling test. These results provide a mathematical characterization of TP-COFs and establish their structural, spectral, and predictive properties within a unified framework.

## Data Availability

The original contributions presented in the study are included in the article/supplementary material, further inquiries can be directed to the corresponding author.
